# Conceptual but not perceptual encoding leads to age differences in priming

**DOI:** 10.3758/s13421-026-01873-7

**Published:** 2026-04-01

**Authors:** Maryam A. Al-Abdulla, Emma V. Ward

**Affiliations:** 1 Department of Social Sciences, Qatar University, Doha, Qatar; 2 Department of Psychology, Faculty of Science and Technology, Middlesex University, London, NW4 4BT UK

**Keywords:** Aging, Priming, Implicit memory, Encoding, Explicit memory

## Abstract

The specific conditions in which age differences in priming emerge remain to be clarified. Three experiments systematically investigated how cognitive processing requirements moderate age differences in priming. Experiment 1 manipulated perceptual/conceptual encoding prior to a continuous identification priming task, and Experiments 2A and 2B manipulated perceptual/conceptual encoding prior to identification (perceptual) and category verification (conceptual) priming tasks. Recognition was also captured to allow comparison of effects on explicit and implicit memory. A further aim was to provide the first direct comparison of online versus laboratory experiments concerned with priming in aging, necessary to understand the feasibility and robustness of online methods, which are increasing in popularity. Experiments 1 (35 young participants, *M*_age_ = 20.51 years; 35 older participants, *M*_age_ = 70.49 years) and 2A (48 young participants, *M*_age_ = 22.65 years; 48 older participants, *M*_age_ = 69.19 years) were conducted online, and Experiment 2B (48 young participants, *M*_age_ = 22.17 years; 48 older participants, *M*_age_ = 76.29 years) was conducted in the laboratory. In all experiments, there was an age difference in perceptual identification priming favouring young adults when encoding was conceptual, but no age difference following perceptual encoding. There was no priming on the category verification task. Priming effects in Experiment 1 were mirrored in the recognition data—recognition was greater in young than older adults following conceptual encoding. There was a reliable age difference in recognition in Experiment 2B, but not Experiment 2A, and no interaction with processing. Findings suggests that age differences in priming emerge as a function of the way stimuli are processed during encoding.

**Supplementary Information:**

The online version contains supplementary material available at 10.3758/s13421-026-01873-7.

Reductions in explicit (declarative) memory with age are well-established. However, the decline versus stability of implicit (nondeclarative) memory with age is debated. Explicit memory involves the conscious retrieval of stored information, while implicit memory is evident when prior exposure to information affects performance on tasks that do not require conscious recollection of that information. For instance, following an encoding phase in which participants are presented with stimuli, explicit memory may be assessed by asking them to differentiate between previously studied (old) and new items in a recognition test. Implicit memory, on the other hand, is assessed indirectly. A common test is perceptual identification, in which participants identify (i.e., name) items that appear briefly or in a degraded form, and priming is evident when stimuli from the encoding phase are identified more quickly or accurately than new items.

Prior studies investigating the effect of aging on implicit memory have produced conflicting results. Null age differences have been reported on tests including word-stem completion (e.g., Jelicic et al., 1996; Light & Singh, 1987; Mitchell & Bruss, 2003; Park & Shaw, 1992; Spaan & Raaijmarkers, 2010), word-fragment completion (e.g., Light et al., 1986; Mitchell & Bruss, 2003), perceptual identification (e.g., Henson et al., 2016; Light et al., 1992; Light & Singh, 1987; Sullivan et al., 1995; Wiggs et al., 2006), picture naming (e.g., Mitchell, 1989; Mitchell & Bruss, 2003; Mitchell et al., 1990; Mitchell & Schmitt, 2006), lexical decision (e.g., Karavanidis et al., 1993; Moscovitch, 1982), homophone spelling (e.g., Howard, 1988), and category exemplar generation (e.g., Isingrini et al., 1995; Light et al., 2000; Mitchell & Bruss, 2003). However, numerous other studies have reported reduced priming in older compared with young adults, including on tests of word-stem completion (e.g., Chiarello & Hoyer, 1988; Davis et al., 1990; Small et al., 1995), perceptual identification (e.g., Abbenhuis et al., 1990; Russo & Parkin, 1993; Ward & Shanks, 2018, 2023, 2024; Ward et al., 2013a, b, 2020), unfamiliar word/object naming (e.g., Keane et al., 2004; Soldan et al., 2009; Wiggs & Martin, 1994), category exemplar generation (e.g., Stuart et al., 2006), category verification (e.g., Light et al., 2000; Ward, 2023), and homophone spelling (e.g., Davis et al., 1990; Howard, 1988; Rose et al., 1986). To account for the discrepancies, reviews by Mitchell and Bruss (2003), Buchner and Wippich (2000), Fleischman (2007), and Ward and Shanks (2018) have concentrated on methodological issues including sample sizes, statistical power, task reliability, and explicit contamination. Each of these issues has been extensively studied, but another factor that may influence whether age differences emerge on a given priming test is the cognitive processing requirements. This issue has not been thoroughly investigated.

Age differences in priming may depend upon the type of processing required during the initial encoding of stimuli—specifically, the extent to which the encoding task relies upon conceptual (meaning-based) processing. While semantic knowledge generally increases with age, as seen, for example, in older adults’ superior vocabulary compared with young adults (e.g., Park et al., 2002; Park & Reuter-Lorenz, 2009), the ability to engage in elaborate semantic cognitive processing while performing a task declines with age (reviewed in Wlotko et al., 2010). Several studies show poorer performance in older than younger adults on tasks that require semantic processing. For example, Zhu et al. (2018) found reduced accuracy in semantic acceptability judgements in older than young adults, and event related potential (ERP) analysis showed reduced activity at the N400, representing semantic processing, in older adults. This age difference in semantic processing has been documented in a meta-analysis by Hoffman and Morcom (2018) and systematic review by Joyal et al. (2022). Joyal et al. (2022) reviewed behavioural and neural indices on a range of tasks involving semantic processing and found reduced accuracy, slower response times, and a reduced N400 ERP effect in older compared with young adults, and in individuals with Alzheimer’s disease compared with healthy older adults. Further, the processing deficit hypothesis (Eysenck, 1974) suggests that older adults are less effective at encoding information when the task involves conceptual processing, so age differences in memory will increase as the depth of processing required by the encoding task increases. Indeed, a number of studies have shown that young adults benefit more from conceptual encoding than older adults: Eysenck (1974) reported greater recall in young than older adults only following semantic encoding (see also Mason, 1979; Simon, 1979), and Mitchell and Perlmutter (1986) found greater recall and recognition in young than older adults only when encoding was intentional, which they argued reflected the more effective use of semantic strategies by young adults.

Despite the conceptual processing deficit in aging, prior studies looking at age differences in priming have varied considerably in the extent to which the encoding task relied upon such processes. Some studies required participants to categorise stimuli, make preference judgements or relative size judgements, while others engaged participants with stimuli in a purely perceptual manner, such as by asking them to make judgements about shape or orientation. Yet other studies have simply presented participants with stimuli, with no way of knowing the processing strategy adopted. This may explain the plethora of mixed findings, but surprisingly few studies have directly manipulated processing to examine this. Recently, Ward (2024) presented young and older participants with object–word pairs under instructions to make perceptual (left/right rotation) or conceptual (natural/manufactured) decisions about the object or word. In a subsequent continuous identification with recognition (CID-R) task participants identified an object (attended, unattended, new) on each trial (priming measure) before making a recognition judgement. Priming and recognition were greater in young than older adults for attended objects, with a larger effect size for conceptual than perceptual items. Stuart et al. (2006) previously published similar findings, in which groups of young and older participants counted vowels (perceptual) or made preference judgements (conceptual) during encoding of words. Priming was reduced by age in the conceptual but not the perceptual condition; however, participants who encoded perceptually performed a perceptual priming task (word-stem completion), and those who encoded conceptually performed a conceptual priming task (category exemplar generation), making it unclear whether the age difference was a function of the type of processing at encoding or test.

Processing requirements at test are thus another important consideration. While perceptual priming tasks, such as perceptual identification, draw upon perceptual processes, conceptual tasks involve semantic processing. For example, in a typical category verification task, participants judge whether presented items match category cues (e.g., shirt–clothing). Priming is evident in faster responses to items shown in a prior encoding phase compared to new items. Like perceptual priming measures, this task captures priming for specific previously studied items, but the decision—judging whether an item is an instance of a particular category—involves conceptual processing (Light et al., 2000). Ward (2023) is the only study to have crossed processing at encoding and test. Participants were presented with words and made letter judgements (perceptual) and living/nonliving judgements (conceptual) prior to identification and category verification priming tests. Priming was greater in young than older adults on both tests, but only when encoding was perceptual. In contrast to the studies above, there were no age differences in priming following conceptual encoding. However, there may have been issues with the encoding manipulation—young adults were significantly slower in the perceptual than the conceptual encoding block, yet one would generally expect faster responses for perceptual judgements than those requiring more elaborate processing. This may suggest that the young participants engaged in additional processing of perceptual items, which could have heightened their priming and exaggerated the age difference in this condition.

## Current investigation

Processing requirements have varied considerably in past studies looking at how aging affects implicit memory, and may have contributed to the mixed findings. The specific conditions in which age differences in priming emerge remain to be clarified. This carries implications for both practice and theory. For example, it has been argued that preserved priming in normal aging may provide a valuable diagnostic tool for Alzheimer’s disease (e.g., Fleischman, 2007), or be used to aid everyday learning such as medication routines or face–name pairs (e.g., Haslam et al., 2011), but this relies on understanding the conditions in which priming is affected by aging. Further, the fate of implicit memory in aging holds implications for understanding the structure of memory (e.g., Gabrieli, 1998; Mitchell, 1989; Schacter & Tulving, 1994; Squire, 2004). We report three experiments that examined how processing interacts with age differences in priming. We considered (1) the way in which processing was manipulated—Experiment 1 manipulated perceptual/conceptual processing at encoding, and Experiments 2A and 2B varied perceptual/conceptual processing at encoding and test, and (2) the mode of testing—online versus laboratory. Experimental research is increasingly being conducted online, providing an excellent way of accessing samples, particularly individuals who are unable to attend in person. Despite significant interest in attempting to understand the reliability and robustness of online experiments versus those conducted in a laboratory setting, there are very few direct comparisons of online versus laboratory studies concerning cognitive aging (for a discussion, see Greene & Naveh-Benjamin, 2022). To provide such a comparison, Experiments 1 and 2A were conducted online, and Experiment 2B was an in-person replication. To our knowledge, this is the first such comparison concerned with priming in aging. Finally, in all experiments, recognition was captured in addition to priming to allow comparison of effects on explicit and implicit memory.

## Experiment 1

Participants completed two encoding-test blocks. During encoding they were presented with objects and in one block made conceptual (natural/manufactured) judgements, and in the other made perceptual (upright/tilted) judgements. Priming and recognition were tested after each encoding block using a CID-R task in which participants identified an object as quickly as possible on each trial before making a recognition judgement. Half of the trials involved studied items, and half involved new items. Methods were like Ward (2024), but there was no simultaneous attention manipulation—a single object was presented on each encoding-phase trial rather than an object–word pair. This change was made because there was no priming for unattended items in Ward (2024). Further, while Ward (2024) was conducted in person, this experiment extended to a controlled online environment. The task was constructed using Gorilla Experiment Builder (www.gorilla.sc), and participants completed it at home using their own computer. Gorilla is now used extensively across different research fields, and has a demonstrated capability of running sensitive RT experiments and replicating well-known psychological effects (e.g., Anwyl-Irvine et al., 2020, 2021; Felisatti et al., 2024; Tomczak et al., 2023). The use of Gorilla has been cited in now thousands of peer-reviewed publications (see https://gorilla.sc/success/publications/). Detailed instructions were provided to participants both in terms of how to perform the tasks and the conditions under which the experiment should be attempted, including (1) in a private space, free of distractions such as phones and television; (2) when they had sufficient time and would not be disturbed as it cannot be paused or restarted; and (3) reading all instructions carefully and only progressing when they were confident that they understood. We predicted main effects of age (young > older on both priming and recognition) and encoding type (recognition: conceptual > perceptual, priming: conceptual ≠ perceptual), and an Age × Encoding-Type interaction (recognition: age difference conceptual condition > age difference perceptual condition, priming: age difference conceptual condition ≠ age difference perceptual condition).

### Method

#### Transparency and data availability

All experiments were pre-registered on the Open Science Framework (Experiment 1: https://osf.io/uz2ka; Experiment 2A: https://osf.io/cde6f; Experiment 2B: https://osf.io/yd9bw), and the de-identified raw data and analysis files are available online (https://osf.io/t7b2y/).

#### Participants

Ethical approval for this and subsequent experiments was granted by the Middlesex University Research Ethics Committee. Sample sizes for all experiments were calculated using G*Power, with *f* = 0.15 and α = .05. Seventy participants were required in Experiment 1, and additional participants were only tested to replace any who were excluded (see Results). The final sample comprised 35 young adults aged 18 to 30 years and 35 older adults aged 65 to 87 years. Young adults were students from Middlesex University, and older adults were from the University of the Third Age (U3A). Data collection took place between 2021 and 2022. Participants were rewarded with course credit (students only) or payment at a rate of £9 per hour. Eligibility requirements included normal/corrected vision, fluency in reading and writing in English, and no cognitive impairment.^1^ Participants were required to tick boxes at the start of the online experiment to provide consent and confirm that they met the eligibility criteria. Background information was then collected prior to the experimental task, and participants completed the multiple-choice section of the Mill Hill Vocabulary Test (Raven et al., 1988; Table 1).

**Table 1:** Participant characteristics in Experiments 1, 2A, and 2B

	Experiments 1		Experiment 2A		Experiment 2B	
	Young *M* (*SD*)	Older *M* (*SD*)	Young *M* (*SD*)	Older *M* (*SD*)	Young *M* (*SD*)	Older *M* (*SD*)
Age (years)	20.51 (3.05)	70.49 (4.84)	22.65 (3.49)	69.19 (4.79)	22.17 (3.28)	76.29 (5.12)
Men/Women (*N*) Education (years)	6/29 14.89 (1.57)	17/18 15.26 (2.73)	17/31 16.08 (2.20)	28/20 15.85 (3.07)	9/39 16.21 (2.36)	15/33 16.33 (3.06)
Self-report Health (*N*) Excellent Good Adequate Poor Extremely poor Mill Hill* Visual acuity* WAIS-III Digit Symbol* WTAR* MMSE	16 14 5 0 0 15.83 (5.20) – – – –	9 19 6 1 0 24.20 (4.06) – – – –	20 26 2 0 0 17.46 (5.34) – – – –	4 29 13 2 0 23.58 (4.02) – – – –	22 25 1 0 0 – 34.67 (6.25) 78.02 (16.29) 43.23 (5.99) –	12 33 3 0 0 – 51.92 (31.39) 60.44 (10.66) 48.54 (2.08) 28.23 (1.59)

*Note.* Mill Hill: The multiple-choice part of the Mill Hill Vocabulary Test (Raven et al., 1988), score range 0–33. Visual acuity: Near Vision Test card (Schneider, 2002), score range: 16 (highest acuity)–160 (lowest acuity). WAIS-III Digit Symbol Substitution Test (Wechsler, 1997), score range 0–133 (used to measure processing speed). WTAR: Wechsler Test of Adult Reading (Wechsler, 2001), score range 0–50. MMSE: Mini Mental State Exam (Folstein et al., 1975), score range 0–30. An MMSE score of <24 indicates probable cognitive impairment, but in Experiment 2b no participant scored below 28

^*^Significant difference between age groups, *p* < .05

#### Design

A mixed-factorial design was used with the between-subjects factor age (young/older adult) and within-subjects factor encoding type (perceptual/conceptual processing). Priming (RTs) and recognition (*d*′) were captured by the CID-R task.

#### Stimuli

**Fig. 1:**
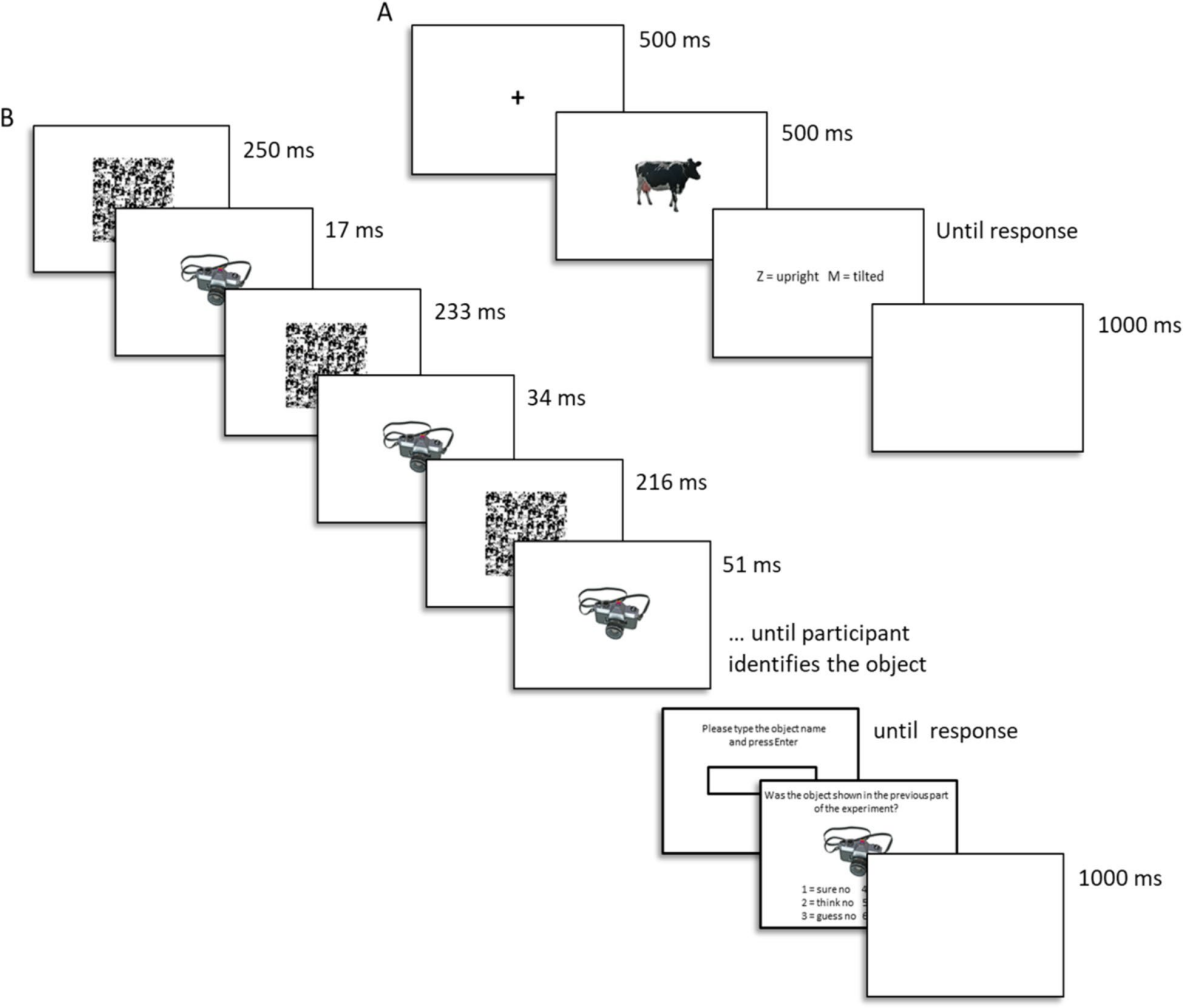
A Trial events in the encoding phase. Participants judged whether objects were upright or tilted (perceptual processing), or natural or manufactured (conceptual processing). **B** Trial events in the CID-R test phase. An object—previously studied or new—gradually clarified from a background mask, and participants identified the item as quickly as possible (priming measure) before making a recognition judgement

Stimuli were 160 colour photographs of objects taken from the Bank of Standardized Stimuli (Brodeur et al., 2014). Objects (384 × 384 pixels) depicted everyday items, half naturally occurring and half manufactured, presented on a white background with shadows removed. Participants completed the experiment on their own computer so screen size varied, but stimuli were automatically configured by Gorilla to fit within the confines of a zone. Screen resolution varied between 1,189 × 669 and 2,048 × 1,152 pixels. The experiment was divided into two counterbalanced encoding-test blocks, one involving perceptual encoding and one involving conceptual encoding, and four sets of 40 objects were rotated. One set (40 items) was presented in each encoding phase, and two in each test phase (80 items, 40 studied and 40 new). Thus, in total each participant was tested on 40 perceptually encoded items, 40 conceptually encoded items, and 80 new items (see Supplementary Materials Table SM1). Based on Ward (2024), during encoding half of the objects were rotated 15° to the left or right (equally distributed between left/right), and the other half remained upright. Two versions of the stimulus sets were counterbalanced, where tilted items in one set were upright in the other. The mask used in the priming segment was a 384 × 384-pixel grid randomly filled with fragments of objects not included in the main stimulus sets (Fig. 1B).

#### Procedure

Participants performed the task using their own desktop or laptop computer. A link was used to launch the experiment, which was compatible with all common browsers but prohibited the use of tablets and mobile phones. A minimum connection speed of 8 Mbps was required. There were two encoding-test blocks, counterbalanced. On each encoding-phase trial, a fixation cross (‘+’) was presented for 500 ms, followed by an object for 500 ms. In the perceptual condition participants judged whether objects were upright or tilted, and in the conceptual condition they judged whether objects were natural or manufactured. The former decision required participants to engage with perceptual detail concerning orientation of items, while the conceptual judgement required more elaborate appraisal of items to make classifications. To respond, participants used keys Z = *upright/natural* and M = *tilted/manufactured*, and a response probe was presented following the object (Fig. 1A). Participants were instructed to respond as quickly as possible, but the response probe remained on the screen until a decision was made. Following a key press there was a blank interval of 1,000 ms before the next trial.

Following encoding in each block there was a 3-min filler phase in which participants decided whether random numbers presented as a continuous stream were odd or even. The test phase in each block involved a CID-R task. Eighty objects appeared in each CID-R task (40 studied in the encoding phase immediately prior and 40 new), and each trial consisted of an object identification (priming) followed by a recognition judgement. On each trial, a mask was first presented for 250 ms. This was followed by an object for 17 ms (approximate screen refresh rate at 60 Hz), and then the mask again for 233 ms, forming a 250 ms block. The object and mask presentations continued to alternate, with the object duration increasing by 17 ms each time and the mask decreasing by this amount (Fig. 1B). The effect is that the object appears to gradually emerge from behind the mask. Participants were instructed to press the spacebar (RT captured) as soon as they could identify the object, at which point the object disappeared and they were prompted to type its name into a box. Immediately after, the object was presented again for a recognition judgement. If a participant had not identified the object by the time it was fully visible (at 7,000 ms), the priming trial was discarded and the screen automatically advanced to the recognition judgement. For the recognition judgement participants were asked whether they believed the object was shown in the previous part of the experiment, and responded using keys 1 = *sure no*, 2 = *think no*, 3 = *guess no*, 4 = *guess yes*, 5 = *think yes*, and 6 = *sure yes*. They were informed that half of the objects were shown before, and half were new. No time limit was imposed. Following response, a blank screen was presented for 1,000 ms prior to the next trial.

At the end of the experiment participants were asked questions to gauge whether they were aware of the purpose of the identification task or attempted to use an explicit strategy. Questions were adapted from Bowers and Schacter (1990) and included (1) *What do you think was the purpose of the* *task where you identified objects as they gradually emerged?*, (2) *Did you suspect prior to the start of* *this task that you would be tested on your memory of the objects? (Yes/No)*, (3) *Did you try to use your memory of the* *objects to help you in this task? (Yes/No)*, and (4) *If yes,* *do you think this strategy helped you, and how so?* Participants who demonstrated an understanding of the purpose of the identification task and/or stated that they attempted to use their memory of the objects to help them in this task were rated as aware.

### Results

#### Data screening and analysis

There were no deviations from the preregistered analysis plan. Twenty participants were replaced: Four young and 12 older adults failed to complete the encoding phase, and two young and one older adult failed to identify objects in the identification task. One older participant requested the withdrawal of their data. In this and subsequent experiments, an alpha level of .05 was used. Partial eta squared (η_p_^2^) effect sizes are reported for analysis of variance (ANOVA) effects, and Cohen’s *d* for *t* tests (all two-tailed). Analyses were performed using JASP 0.16.2, and Bayes factor analysis was conducted for nonsignificant effects, with BF_10_ values <0.33 considered as support for the null hypothesis (Dienes, 2014).

#### Encoding

The proportion of correct responses and RTs in the conceptual and perceptual encoding conditions is summarised in Table 2. In this and subsequent experiments, no outliers were observed in the RT data and no trials were removed. While accuracy in the conceptual condition was based on the correct answer (i.e., natural/manufactured item), accuracy in the perceptual condition was calculated based on the modal response across participants, given possible subjectivity in the upright/tilted judgements.^2^ Thus, if the majority of participants judged a particular object as upright, then this was considered the correct answer. A 2 (age) × 2 (encoding type) mixed ANOVA on accuracy revealed main effects of encoding type, *F*(1,68) = 222.98, *p* < .001, η_p_^2^ = 0.77, with greater accuracy in the conceptual (marginal mean = 91.75%) than the perceptual (marginal mean = 76.25%) condition, and age, *F*(1,68) = 8.73, *p* = .004, η_p_^2^ = 0.11, with greater accuracy in young (marginal mean = 85.63%) than older (marginal mean = 82.36%) adults, but no interaction, *F*(1,68) = 0.38, *p* = .538, η_p_^2^ = 0.01 (BF_10_ = 0.29). On RTs there was no main effect of encoding type, *F*(1,68) = 1.34, *p* = .251, η_p_^2^ = 0.02 (BF_10_ = 0.32), or age, *F*(1,68) = 0.02, *p* = .903, η_p_^2^ < .001 (BF_10_ = 0.31), and no interaction, *F*(1,68) = 0.70, *p* = .405, η_p_^2^ = 0.01 (BF_10_ = 0.33).

**Table 2:** Performance in the encoding phase in Experiments 1, 2A and 2B

	Experiments 1		Experiment 2A		Experiment 2B	
	Young	Older	Young	Older	Young	Older
	*M* (*SD*)	*M* (*SD*)	*M* (*SD*)	*M* (*SD*)	*M* (*SD*)	*M* (*SD*)
Accuracy (%)						
Perceptual	78.21 (5.99)	74.29 (6.17)	73.07 (9.54)	82.97 (9.67)	64.22 (15.98)	58.23 (14.40)
Conceptual	93.07 (5.94)	90.43 (7.26)	89.74 (6.19)	86.82 (9.35)	96.25 (4.19)	87.76 (8.82)
RTs (ms)						
Perceptual	979 (368)	1004 (492)	953 (269)	860 (203)	1262 (409)	1008 (467)
Conceptual	966 (291)	922 (285)	792 (234)	816 (195)	1057 (282)	996 (410)

#### Priming

**Fig. 2:**
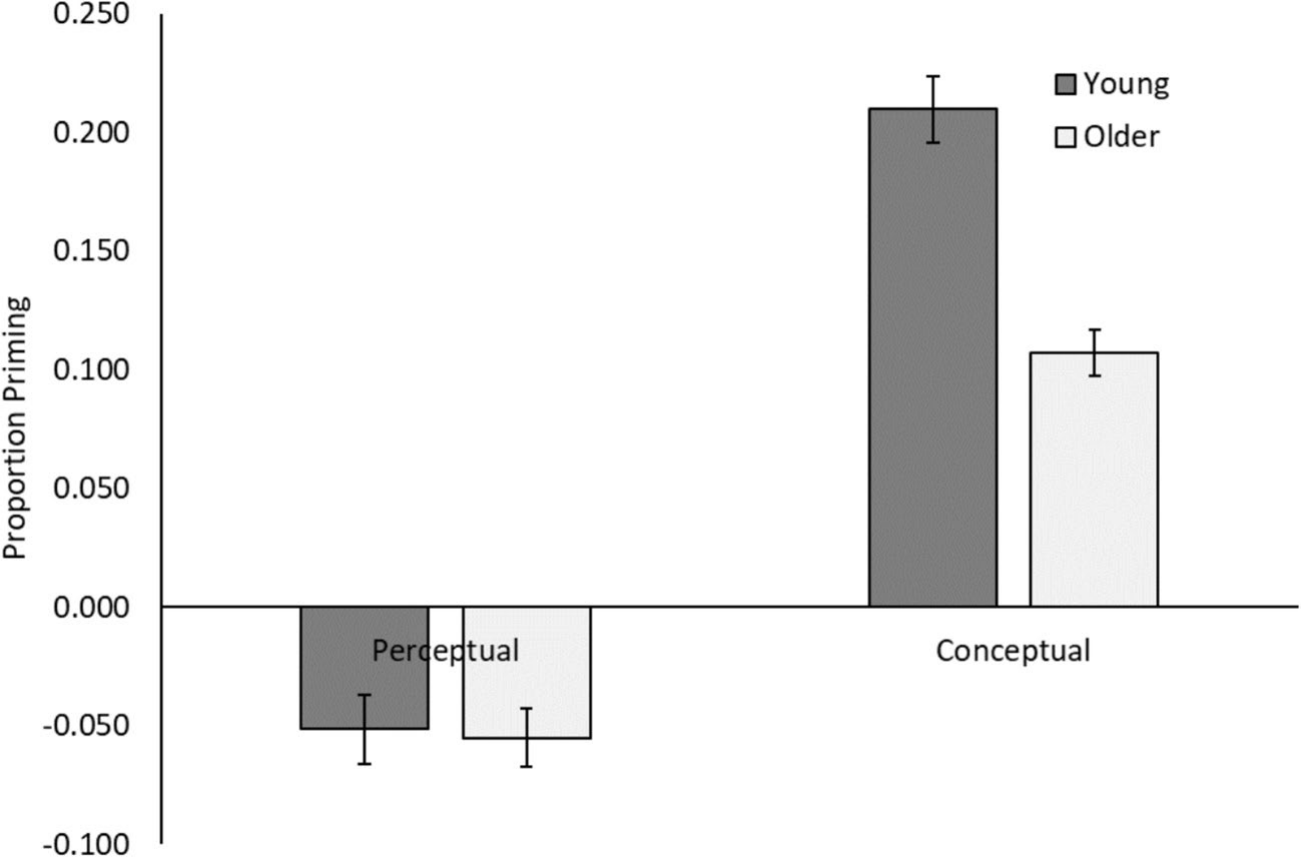
Priming in young and older adults following conceptual and perceptual encoding in Experiments 1 (standard error bars)

Trials associated with incorrect object identifications and/or RTs <200 ms or >3 standard deviations from the mean were trimmed. Spelling mistakes and typos were permitted, but trials with entirely incorrect object names or random responses were removed. Out of total 11,200 trials, 1,012 were removed (423 from young adults, 589 from older adults). A priming score was calculated for each participant in each condition as (RTnew − RTold)/RTnew (Fig. 2; Table 3 for RTs). A 2 (age) × 2 (encoding type) mixed ANOVA revealed a main effect of encoding type, *F*(1,68) = 343.54, *p* < .001, η_p_^2^ = 0.84, with robust priming in the conceptual condition (marginal mean = 0.16), but no priming in the perceptual condition (marginal mean = −0.05). There was a main effect of age, *F*(1,68) = 14.47, *p* < .001, η_p_^2^ = 0.18, with greater priming in young (marginal mean = 0.08) than older (marginal mean = 0.03) adults, and an Encoding-Type × Age interaction, *F*(1,68) = 18.49, *p* < .001, η_p_^2^ = 0.21. Priming was greater in young than in older adults only in the conceptual condition, *t*(68) = 6.08, *p* < .001, *d* = 1.45 (perceptual condition): *t*(68) = 0.19, *p* = .847, *d* = 0.05, BF_10_ = 0.25.

**Table 3:** Mean priming-task RTs (ms) and priming scores in young and older adults in Experiments 1, 2A, and 2B

	Young	Older
	*M* (*SD*)	*M* (*SD*)
Experiments 1		
Old items		
Perceptual block	2,809 (562)	3,681 (678)
Conceptual block	2,501 (515)	3,315 (616)
New items		
Perceptual block	2,673 (515)	3,501 (673)
Conceptual block	3,157 (556)	3,718 (660)
Priming		
Perceptual encoding	−.051 (.086)	−.055 (.073)
Conceptual encoding	.210 (.081)	.107 (.058)
Experiment 2A		
CID-R task		
Perceptual items (PP)	3,129 (633)	3,435 (727)
Conceptual items (CP)	3,044 (632)	3,454 (758)
New items	3,271 (537)	3,494 (677)
CV-R task		
Perceptual items (PC)	1,684 (399)	2,119 (440)
Conceptual items (CC)	1,654 (375)	2,108 (405)
New items	1,670 (347)	2,100 (403)
Priming		
PP	.046 (.083)	.016 (.101)
CP	.070 (.109)	.009 (.122)
PC	−.015 (.175)	−.009 (.078)
CC	.003 (.163)	−.006 (.070)
Experiment 2B		
CID-R task		
Perceptual items (PP)	1,799 (358)	2,373 (550)
Conceptual items (CP)	1,806 (378)	2,453 (547)
New items	1,897 (379)	2,453 (455)
CV-R task		
Perceptual items (PC)	1,666 (543)	2,773 (643)
Conceptual items (CC)	1,673 (536)	2,785 (655)
New items	1,674 (562)	2,626 (593)
Priming		
PP	.048 (.080)	.033 (.122)
CP	.047 (.072)	.002 (.086)
PC	−.003 (.097)	−.060 (.106)
CC	−.008 (.098)	−.062 (.075)

*Note.* PP: perceptual encoding—perceptual test; CP: conceptual encoding—perceptual test; PC: perceptual encoding—conceptual test; CC: conceptual encoding—conceptual test

#### Recognition

**Fig. 3:**
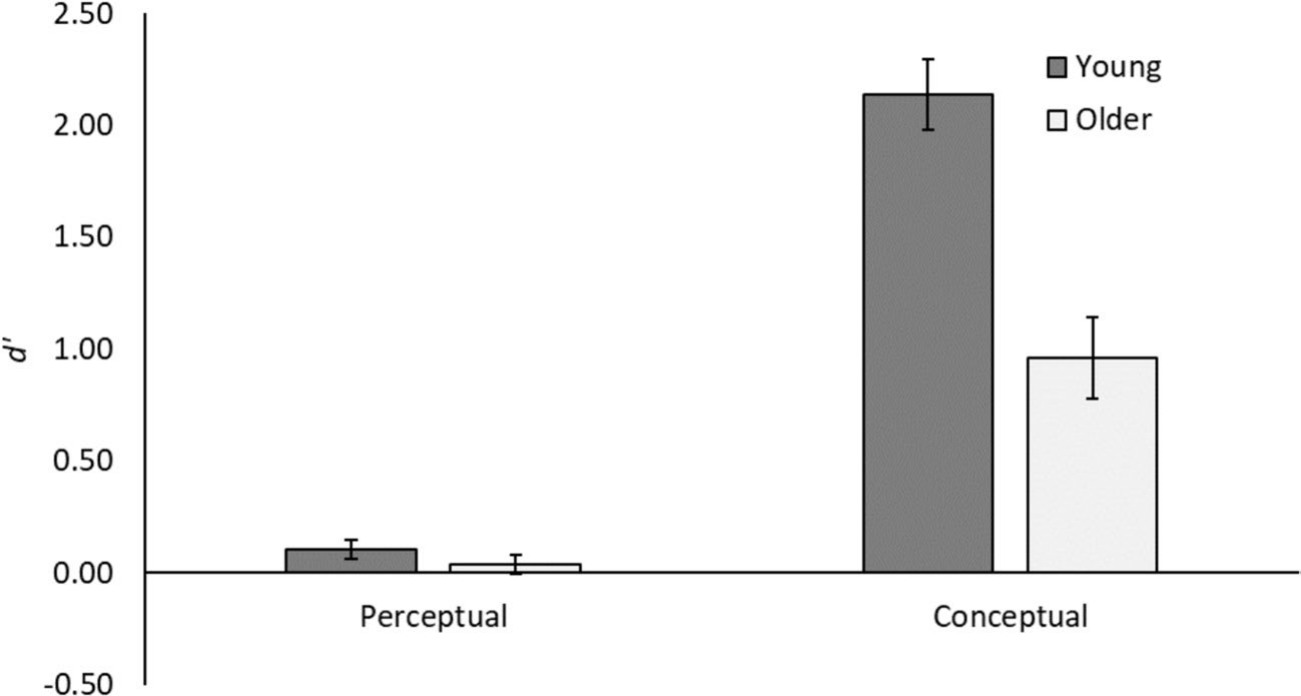
Recognition (*d′*) in young and older adults in Experiment 1 (standard error bars)

Scores on the 6-point scale were collapsed into ‘yes’ (4–6) and ‘no’ (1–3) responses. The scale was used to capture broad responses across different levels of confidence and reduce potential issues with response bias, but analysis of confidence was not possible in any of the experiments because participants did not use all response options. A *d’* score for each participant in each condition was calculated as the *z*-transformed proportion of hits minus false alarms (FA; Fig. 3; Table 4 for proportions of hits and FA; see Supplementary Materials for response bias scores and analysis). The Snodgrass and Corwin (1988) correction was applied to hit and FA rates of 0 or 1: hit rate = [*n* hits + 0.5]/[*n* old + 1]; FA rate = [*n* FAs + 0.5]/[*n* new + 1].

**Table 4:** Proportions of hits and false alarms (FA), and recognition sensitivity (*d*′) in the recognition tasks in Experiment 1, 2A, and 2B

	Young	Older
	*M* (*SD*)	*M* (*SD*)
Experiment 1		
Perceptual block		
Hits	0.58 (0.21)	0.67 (0.32)
FA	0.55 (0.21)	0.66 (0.32)
Conceptual block		
Hits	0.87 (0.12)	0.86 (0.15)
FA	0.25 (0.31)	0.56 (0.41)
*d′*		
Perceptual encoding	0.10 (0.27)	0.04 (0.26)
Conceptual encoding	2.14 (0.93)	0.96 (1.07)
Experiment 2A		
CID-R task		
Perceptual Hits	0.79 (0.18)	0.61 (0.21)
Conceptual Hits	0.90 (0.09)	0.82 (0.14)
FA	0.32 (0.26)	0.19 (0.15)
CV-R Task		
Perceptual Hits	0.77 (0.25)	0.74 (0.20)
Conceptual Hits	0.81 (0.22)	0.78 (0.19)
FA	0.18 (0.24)	0.15 (0.12)
*d′*		
PP	1.46 (0.98)	1.34 (0.78)
CP	1.92 (0.96)	2.03 (0.75)
PC	2.07 (1.14)	1.91 (0.80)
CC	2.21 (1.18)	2.08 (0.85)
Experiment 2B		
CID-R task		
Perceptual Hits	0.87 (0.14)	0.71 (0.22)
Conceptual Hits	0.86 (0.11)	0.74 (0.22)
FA	0.14 (0.12)	0.17 (0.15)
CV-R task		
Perceptual Hits	0.86 (0.09)	0.76 (0.21)
Conceptual Hits	0.84 (0.11)	0.74 (0.19)
FA	0.15 (0.11)	0.19 (0.19)
*d′*		
PP	2.53 (0.70)	1.75 (0.81)
CP	2.43 (0.66)	1.87 (0.78)
PC	2.38 (0.65)	1.87 (0.87)
CC	2.31 (0.65)	1.78 (0.79)

*Note.* Hits: old items judged old; FA: new items judged old. *d′* = zHits − zFA. PP: perceptual encoding—perceptual test; CP: conceptual encoding—perceptual test; PC: perceptual encoding—conceptual test; CC: conceptual encoding—conceptual test

There were main effects of encoding type, *F*(1,68) = 150.06, *p* < .001, η_p_^2^ = 0.69, with greater recognition in the conceptual (marginal mean = 1.55) than the perceptual (marginal mean = 0.07) condition, and age, *F*(1,68) = 23.88, *p* < .001, η_p_^2^ = 0.26, with greater recognition in young (marginal mean = 1.12) than older (marginal mean = 0.50) adults, and an Encoding-Type × Age interaction, *F*(1,68) = 21.32, *p* < .001, η_p_^2^ = 0.24. Recognition was greater in young than older adults when encoding was conceptual, *t*(68) = 4.91, *p* < .001, *d* = 1.18, but there was no reliable age difference following perceptual encoding,* t*(68) = 1.03, *p* = .305, *d* = 0.25 (BF_10_ = 0.39).

#### Awareness questionnaire

Six young and seven older adults were aware that the identification priming task was related to memory. Because the proportion of aware to unaware participants was low, no statistical comparison of priming in these groups was conducted (see Supplementary Materials Table SM2 for means).

### Discussion

Experiment 1 manipulated perceptual/conceptual encoding of objects in young and older adults prior to a CID-R task to assess priming (perceptual identification) and recognition. Both priming and recognition were greater in young than older adults following conceptual encoding, but there were no age differences following perceptual encoding. When encoding was perceptual, recognition was close to zero in both age groups, and priming was below zero. Findings are consistent with Ward (2024), which also used a CID-R task but a different encoding phase involving a simultaneous attention manipulation. In Ward (2024), priming and recognition were greater in young than older adults for attended objects, with a larger effect size in the conceptual than the perceptual condition. The prior study was conducted in a laboratory setting, so the present experiment corroborates and extends the observations to a controlled online environment. However, while Experiment 1 provides evidence for an age difference in perceptual identification priming as a function of conceptual encoding, it does not provide insight into how encoding interacts with age differences on a priming task involving conceptual processing. To address this, Experiment 2A varied perceptual/conceptual processing at both encoding and test.

## Experiment 2A

In Experiment 2A, again conducted online using Gorilla, participants were presented with objects in two counterbalanced encoding blocks and made perceptual (upright/tilted) and conceptual (natural/manufactured) judgements prior to a blocked test phase to measure perceptual priming (CID-R task) and conceptual priming (category verification with recognition, CV-R, task). This allowed comparison of age effects in the following conditions: perceptual encoding—perceptual test [PP], conceptual encoding—perceptual test [CP], perceptual encoding—conceptual test [PC], conceptual encoding—conceptual test [CC]. The perceptual and conceptual priming tasks were matched as far as possible on all characteristics except processing. Both involved a speeded task with RTs as the dependent measure, and within-trial durations were consistent (see Procedure). This experiment was based on Ward’s (2023) registered report study, with the following changes: (1) The objects and encoding phase judgements from Experiment 1 were used (stimuli were the same in all the present experiments to maintain consistency), rather than the word stimuli used in Ward (2023). (2) The identification and category verification priming tasks were the same as in Ward (2023), but we included a concurrent recognition judgement for consistency with Experiment 1 and to allow comparison of effects on explicit and implicit memory. Based on Experiment 1 we predicted main effects of age (young > older) and encoding type (conceptual > perceptual) on both priming and recognition, as well as an Age × Encoding-Type × Test-Type interaction. Specifically, we expected the age difference in priming and recognition to be largest in the CC condition, and smallest in the PP condition.

### Method

#### Participants

A total of 48 young adults aged 18 to 30 years and 48 older adults aged 65 to 86 years participated (Table 1). Data collection took place in 2022. Participants were primarily recruited using Prolific^3^, but the young group also comprised 16 students from Middlesex University. Eligibility criteria were the same as in Experiment 1, and older adults were asked additional questions to screen for cognitive impairment: (1) *Have you ever been diagnosed with cognitive impairment or dementia?*, (2) *Do you have difficulty with remembering names/familiar faces?*, and (3) *Do you have difficulty following conversations? *We planned to exclude participants who responded ‘yes’ to all questions, but no participants responded ‘yes’ to questions 1 and 3. Seven responded ‘yes’ to question 2, but their experimental task performance was comparable to the rest of the sample so we do not believe any participants were cognitively impaired. Further, prescreening restrictions were also embedded into Prolific, such that the experiment was only advertised to prospective participants without cognitive impairment within the desired age groups.

#### Design

A mixed factorial design was used with the between-subjects factor age (young/older adult), and within-subjects factors encoding type (perceptual/conceptual) and test type (perceptual/conceptual). Both priming tasks captured RTs as the dependent measure, and recognition was based on *d′*.

#### Stimuli

The same stimuli from Experiment 1 were used. Eight sets of 20 objects were rotated: two sets (i.e., 40 items) were presented in each encoding phase, and four sets (i.e., 80 items) in each test. Thus, each test included 40 previously studied items, 20 from the perceptual encoding phase and 20 from the conceptual encoding phase, and 40 new items (see Supplementary Materials Table SM1). Two versions of the stimulus sets were created to counterbalance tilted/upright objects at encoding. Participant screen resolution varied between 800 × 400 and 1,920 × 1,080 pixels.

#### Procedure

Participants performed the experiment online using their own computer, with the same restrictions as in Experiment 1. In the encoding phase participants were presented with 80 objects (40 per block), and within-trial events were identical to Experiment 1: A fixation cross was presented for 500 ms, followed by an object for 500 ms. Participants judged whether objects were upright or tilted (perceptual block), or natural or manufactured (conceptual block), using Z = *upright/natural* and M = *tilted/manufactured*. They were asked to respond as quickly as possible, but the response probe remained on the screen until a key press was made. The order of the encoding blocks was counterbalanced between participants.

**Fig. 4:**
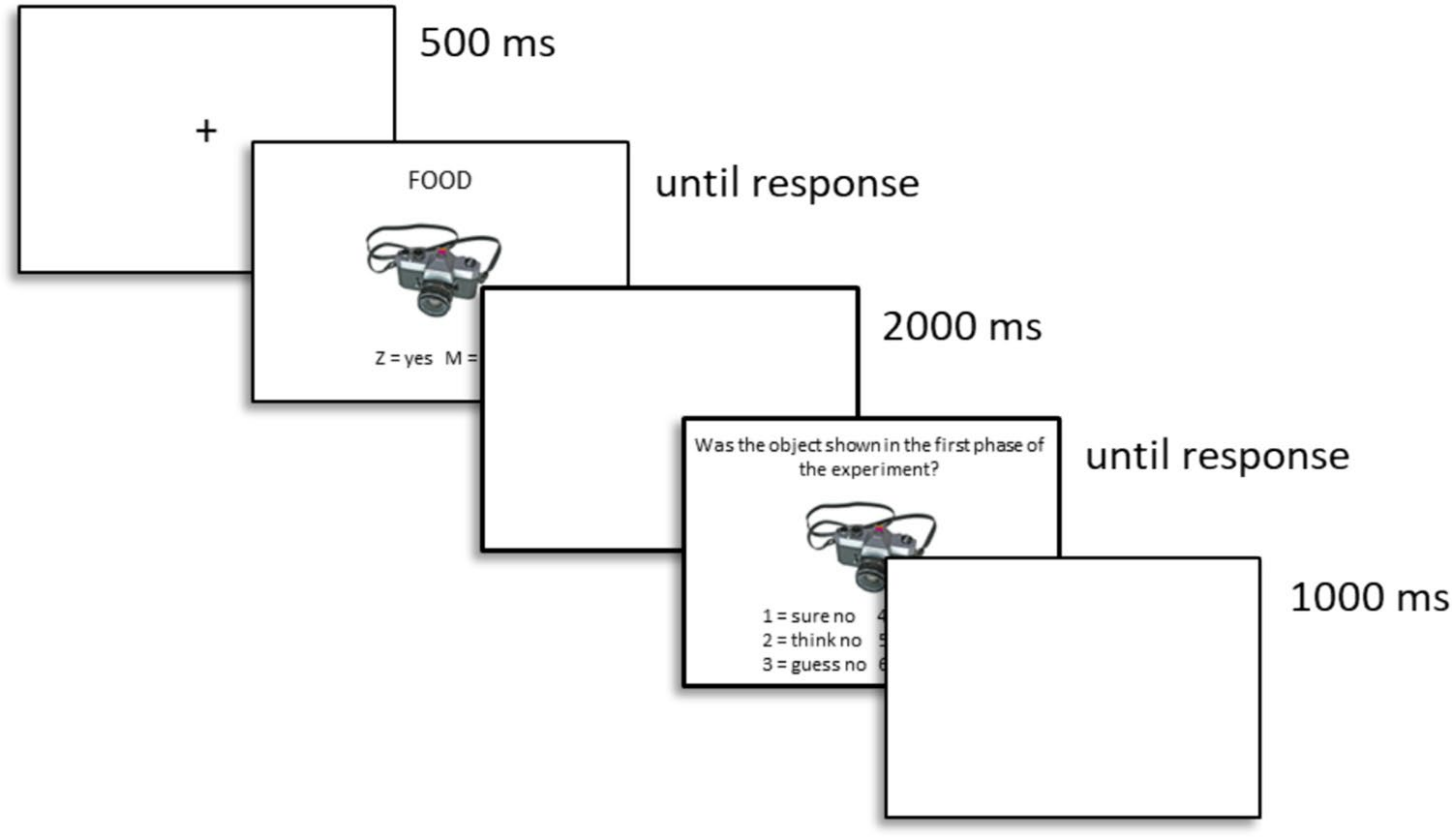
Trial events in the CV-R task. Participants judged as quickly as possible whether an object (studied or new) matched a given category (conceptual priming measure) before making a recognition judgement

Following the same filler phase as Experiment 1, participants performed the test phase, which comprised the perceptual priming task (CID-R) and the conceptual priming task (CV-R). The order of these tasks was determined by encoding: if the perceptual encoding block was performed first, the CID-R task was performed first at test, but if the conceptual encoding block was performed first, the CV-R task was performed first at test. A different 80 objects were presented in each task—40 previously studied (20 from the perceptual encoding block and 20 from the conceptual encoding block) and 40 new. All trial events and timings in the CID-R task were identical to Experiment 1: An object gradually emerged, and participants identified it as quickly as possible before making a recognition judgement. In the CV-R task, participants were informed that their objective was to decide whether objects matched category names as quickly as possible. On each trial a fixation cross was presented for 500 ms followed by an object and category label (e.g., ‘food’), and participants judged whether the object matched the category by selecting Z = *yes* or M = *no* (Fig. 4). For half of the objects, the correct category was presented, and for the other half, an incorrect category, such that the correct response was ‘yes’ for half of all trials. The object and response cue remained on the screen until a key press was made (RT captured), at which point the screen cleared. For consistency with the CID-R task, if no response was made by 7,000 ms, the priming trial was discarded, and the screen automatically advanced to the recognition judgement. Further, to match the timings with the CID-R task as far as possible, a blank screen was presented for 2,000 ms before the object was presented again for the recognition judgement (the period in which participants type their response in the CID-R task).

Following the test phase, participants completed a short awareness questionnaire. They were asked (1) *What do you think was the purpose of the task where you identified objects as they gradually emerged?*, (2) *What do you think was the purpose of the task where you judged if objects matched given categories?*, (3) *Did you suspect prior to the start of these test that you would be tested on your memory of the objects? (Yes/No)*, (4) *Did you try to use your memory of the objects to help you in these tasks? (Yes/No)*, and (5) *If yes, do you think this strategy helped you, and how so?* Participants who demonstrated an understanding of the purpose of one or both tasks, or stated that they attempted to use their memory of the objects to perform the task/s, were rated as aware.

### Results

#### Data screening

The preregistered encoding-phase accuracy threshold was relaxed from 70% to 60%^4^, but there were no other deviations from the analysis plan. Thirty-two participants (13 young, 19 older) were replaced. One young participant had a lack of recorded responses, eight failed to complete the CID-R task, and four achieved <80% correct identifications in the CID-R task. One older participant had a lack of recorded responses in the encoding phase, eight failed to complete the CID-R task, and 10 achieved <80% correct identifications in the CID-R task.

#### Encoding

See Table 2 for the proportions of correct responses and RTs in the conceptual and perceptual encoding blocks. Accuracy in the perceptual block was once again calculated based on the modal response. There were main effects of encoding type, *F*(1,94) = 102.66, *p* < .001, η_p_^2^ = 0.52, with greater accuracy in the conceptual (marginal mean = 88.28%) than the perceptual (marginal mean = 78.02%) condition, and age, *F*(1,94) = 5.52, *p* = .021, η_p_^2^ = 0.06, with greater accuracy in older (marginal mean = 84.90%) than young (marginal mean = 81.41%) adults, and an Encoding-Type × Age interaction, *F*(1,94) = 40.02, *p* < .001, η_p_^2^ = 0.30. Older adults were more accurate than young adults in the perceptual encoding block, *t*(94) = 5.05, *p* < .001, *d* = 1.03, but there was no reliable age difference in accuracy in the conceptual block, *t*(94) = 1.80, *p* = .075, *d* = 0.37 (BF_10_ = 0.89). On RTs there was a main effect of encoding type, *F*(1,94) = 35.06, *p* < .001, η_p_^2^ = 0.27, with faster responses in the conceptual (marginal mean = 804 ms) than the perceptual (marginal mean = 906 ms) condition. There was no main effect of age, *F*(1,94) = 0.65, *p* = .421, η_p_^2^ = 0.01 (BF_10_ = 0.38), but an Encoding-Type × Age interaction, *F*(1,94) = 11.36, *p* = .001, η_p_^2^ = 0.11. Both groups were quicker to respond in the conceptual than the perceptual block, with a larger effect size in young adults, young: *t*(47) = 5.55, *p* < .001, *d* = 0.80; older: *t*(47) = 2.34, *p* = .024, *d* = 0.34.

#### Priming

**Fig. 5:**
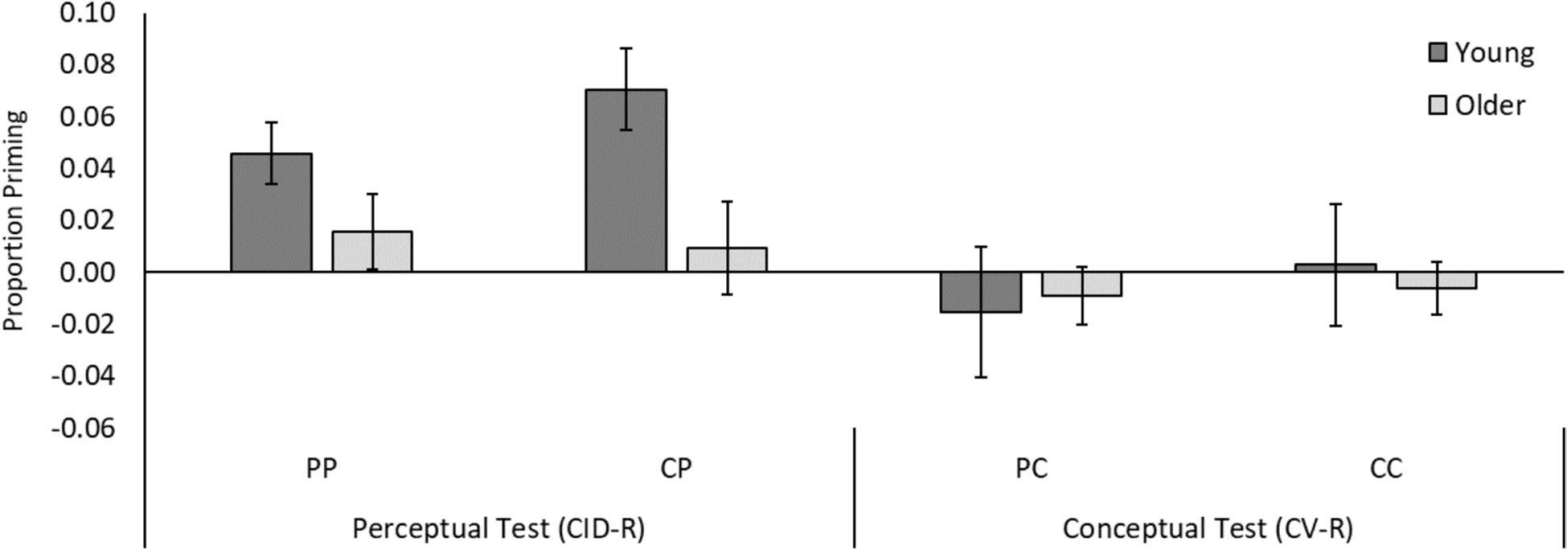
Priming in young and older adults in Experiment 2A (standard error bars). PP: perceptual encoding—perceptual test; CP: conceptual encoding—perceptual test; PC: perceptual encoding—conceptual test; CC: conceptual encoding—conceptual test

Trials associated with incorrect object identifications (CID-R task) or incorrect category judgements (CV-R task) were removed, as well as trials with RTs <200 ms or >3 standard deviations from the participants’ mean. A total of 992/7680 trials were removed from the CID-R task (young = 357; older = 635), and 601/7680 trials were removed from the CV-R task (young = 318; older = 283). Priming scores were calculated as in Experiment 1 (Fig. 5; Table 3 for RTs). Priming was absent in both groups on the CV-R test, so these scores were not included in the analysis. A 2 (age) × 2 (encoding type) ANOVA on CID-R priming revealed a main effect of age, *F*(1,94) = 6.49, *p* = .012, η_p_^2^ = 0.07, with greater priming in young (marginal mean = 0.06) than older (marginal mean = 0.01) adults. There was no main effect of encoding type, *F*(1,94) = 0.66, *p* = .418, η_p_^2^ = 0.07 (BF_10_ = 0.21), and no interaction, *F*(1,94) = 1.66, *p* = .200, η_p_^2^ = .017 (BF_10_ = 1.49). A priori planned comparisons of priming in young and older adults in the PP and CP conditions (akin to the perceptual and conceptual conditions in Experiment 1) revealed significantly greater priming in young than in older adults following conceptual encoding (CP condition), *t*(94) = 2.56, *p* = .012, *d* = 0.52, but no reliable age difference following perceptual encoding (PP condition), *t*(94) = 1.61, *p* = .112, *d* = 0.33 (BF_10_ = 0.67).

#### Recognition

**Fig. 6:**
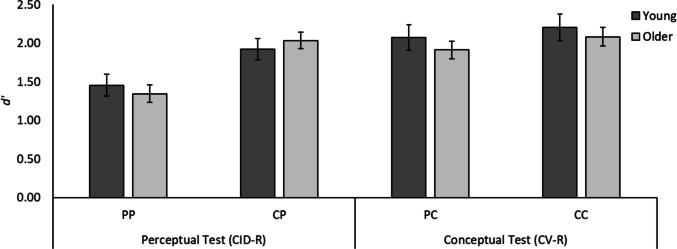
Recognition in young and older adults in the four conditions in Experiment 2A (standard error bars)

We calculated *d′* as in Experiment 1 (Fig. 6; Table 4 for a breakdown of responses). It should be noted that the recognition task in the CID-R and CV-R tests was identical, but the data are presented in the same way as priming for ease of comparison. A 2 (age) × 2 (encoding type) × 2 (test type) ANOVA revealed main effects of encoding type, *F*(1,94) = 55.03, *p* < .001, η_p_^2^ = 0.37, with greater recognition in the conceptual (marginal mean = 2.06) than the perceptual (marginal mean = 1.70) condition, and test type, *F*(1,94) = 17.22, *p* < .001, η_p_^2^ = 0.16, with greater recognition on the CV-R (marginal mean = 2.07) than the CID-R (marginal mean = 1.69) task, but no main effect of age, *F*(1,94) = 0.20, *p* = .658, η_p_^2^ = .002 (BF_10_ = 0.31). There was an Encoding-Type × Test-Type interaction, *F*(1,94) = 31.26, *p* < .001, η_p_^2^ = 0.25, and follow-up tests revealed greater recognition following conceptual than perceptual encoding on both the CID-R test, *F*(1,94) = 90.89, *p* < .001, η_p_^2^ = 0.49 (marginal means: conceptual = 1.98; perceptual = 1.40) and the CV-R test, *F*(1,94) = 5.67, *p* = .019, η_p_^2^ = 0.06 (marginal means: conceptual = 2.14; perceptual) = 1.99), but a larger effect size in the former. There were no other interactions, Encoding Type × Age: *F*(1,94) = 1.78, *p* = .185, η_p_^2^ = 0.02 [BF_10_ = 0.35]; Test Type × Age: *F*(1,94) = 0.61, *p* = .439, η_p_^2^ = 0.01 [BF_10_ = 0.32]; Encoding Type × Test Type × Age: *F*(1,94) = 1.53, *p* = .219, η_p_^2^ = 0.02 [BF_10_ = 0.56].

#### Awareness questionnaire

Nineteen young and eight older adults were aware of the purpose of the identification priming task, but only six young and three older adults were rated aware in the category verification task. Given that 40% of young participants were aware in the identification task, a 2 (encoding type) × 2 (awareness) ANOVA was conducted on CID-R priming scores, showing no main effect of encoding type, *F*(1,46) = 1.70, *p* = .199, η_p_^2^ = 0.04 (BF_10_ = 0.65), or awareness, *F*(1,46) = 2.95, *p =* .093, η_p_^2^ = 0.06 (BF_10_ = 0.97), and no interaction, *F*(1,46) = 1.63, *p* = .208, η_p_^2^ = 0.03 (BF_10_ = 0.59). No other statistical comparisons were performed due to the low levels of awareness (see Supplementary Materials Table SM2 for mean priming in aware and unaware participants).

### Discussion

There was no priming in either age group on the CV-R task (conceptual priming). On the CID-R task (perceptual priming), young adults produced significantly greater priming than older adults when encoding was conceptual—that is, in the CP condition. This is consistent with Experiment 1. These observations, however, are different to those in Ward (2023), where a reliable age difference in priming emerged on the CID-R and CV-R priming tasks only following perceptual encoding. Ward (2023) was also conducted online, but there were a few key differences: The present experiment used the object stimuli and encoding phase judgements from Experiment 1, while the earlier study used concrete nouns. In the perceptual encoding block participants judged whether the first and last letter were in alphabetical order, and in the conceptual block they judged whether words depicted living or non-living things. Further, the present experiment incorporated a recognition judgement at test. It is possible that this could have interfered with one or both priming measures to give rise to the different outcomes, but this is unlikely given evidence that the CID-R task and similar speeded measures are unaffected by the presence of a concurrent recognition judgement (e.g., Brown et al., 1991, 1996; Ward et al., 2013a; see General Discussion). Further, we can be confident that priming in this experiment was not affected by explicit contamination because awareness levels were generally low, and statistical comparison of priming in aware and unaware young participants on the CID-R task revealed no difference. It is more likely that the different findings are due to the different stimuli and/or encoding-phase judgements.

Recognition was greater following conceptual than perceptual encoding, and on the CV-R than the CID-R test (see General Discussion), but did not differ between young and older adults. While age differences on recognition tasks are often smaller than on other explicit tests such as recall (e.g., Craik & McDowd, 1987; Danckert & Craik, 2013; Light & La Voie, 1993; Moscovitch & Winocur, 1992; Nyberg et al., 2003; Rhodes et al., 2019; Whiting & Smith, 1997), based on Experiment 1 we expected a reliable age difference following conceptual encoding. It is noteworthy that young adults’ recognition following conceptual encoding in Experiment 1 (*M*=2.14) was greater than in the equivalent condition (CP) in Experiment 2A (*M* = 1.92), but the opposite was true in older adults (Experiment 1 *M* = 0.96, Experiment 2A *M* = 2.03). We believe, based on close screening of the data, that participants performed the task diligently, but cannot rule out that there may have been a higher proportion of young participants putting in lower effort in Experiment 2A, or that the older participants were particularly high functioning. It is also interesting that recognition for perceptually encoded items was close to zero in Experiment 1, but more robust in the equivalent condition (PP) in Experiment 2A. The precise reasons are unclear, but could reflect differences in the samples (students [young] and U3A members [older] in Experiment 1 versus Prolific.co users in Experiment 2A). There were no noteworthy differences in age or other background characteristics, except fewer men in Experiment 1, but we cannot rule out differences in other unmeasured factors.

## Experiment 2B

Experiment 2B was a replication of Experiment 2A, conducted in the laboratory. As outlined previously, online experimental research is rapidly gaining popularity, and there is considerable interest in establishing the reliability and robustness compared with traditional laboratory experiments. There is growing evidence that the Gorilla platform is capable of running sensitive online experiments and replicating well-known effects (e.g., Anwyl-Irvine et al., 2020, 2021; Felisatti et al., 2024; Tomczak et al., 2023), but there are few direct comparisons of online versus laboratory experiments in the field of cognitive aging. This is the first such comparison concerned with priming in aging. Predictions were the same as in Experiment 2A.

### Method

#### Participants and design

Data collection took place between May and November 2022 in London, UK. The design was identical to Experiment 2A. Forty-eight young adults (18 to 31 years) and 48 older adults (67 to 90 years) participated. Young participants were students from Middlesex University, and older participants were from the U3A. Eligibility criteria were the same as in Experiment 1 and 2A. Older participants were screened for cognitive impairment using the Mini Mental State Exam (Folstein et al., 1975), and formal tests of visual acuity (Near Vision Test; Schneider, 2002), processing speed (Wechsler Adult Intelligence Scale, Digit Symbol Substitution subtest; Wechsler, 1997), and intelligence (Wechsler Test of Adult Reading; Wechsler, 2001) were administered on all participants (Table 1).

#### Stimuli and procedure

The same stimuli were used once more. The experiment was developed using E-Prime 2.0, and participants performed the task at Middlesex University in a private, sound dampened cubicle on a PC with a screen resolution of 1,920 × 1,080 pixels. The task was identical to Experiment 2A, except that the encoding-phase judgement in the perceptual block was changed to angular/rounded and all objects were presented upright. Participants in Experiment 1 and 2A appeared to struggle with the upright/tilted judgement—response times were generally slower in the perceptual than the conceptual encoding block, and accuracy was lower, particularly in young adults. In this experiment the correct answer (angular/rounded) was determined by an independent sample of 12 volunteers (*M*_age_ = 23.15 years, *SD* = 4.69) who rated whether each of the 160 objects was angular or rounded. Classification agreement was high for all except for two items, which were replaced, and the modal response of the participants in Experiment 2B matched the volunteers’ classifications. All trial events and timings in the encoding and test phases were as described in Experiment 2A. On completion of the experiment participants were given the same awareness questionnaire, and finally performed the background and screening tests.

### Results

#### Data screening

The preregistered encoding-phase accuracy threshold once again needed to be relaxed (see Footnote 4), but there were no other deviations from the analysis plan. No participants were replaced.

#### Encoding phase

See Table 2 for mean accuracy and RTs. A 2 (age) × 2 (encoding type) ANOVA on accuracy revealed main effects of encoding type, *F*(1,94) = 295.94, *p* < .001, η_p_^2^ = 0.76, with greater accuracy in the conceptual (marginal mean = 92.01%) than the perceptual (marginal mean = 61.22%) condition, and age, *F*(1,94) = 20.07, *p* < .001, η_p_^2^ = 0.18, with greater accuracy in young (marginal mean = 80.23%) than older (marginal mean = 72.99%) adults, but no interaction, *F*(1,94) = 0.49, *p* = .487, η_p_^2^ < 0.01 (BF_10_ = 0.30). On RTs there was a main effect of encoding type, *F*(1,94) = 4.68, *p* = .033, η_p_^2^ = 0.05, with faster responses in the conceptual (marginal mean = 1,026 ms) than the perceptual (marginal mean = 1,135 ms) condition, and a main effect of age, *F*(1,94) = 6.10, *p =* .015, η_p_^2^ = 0.06, with faster responses in older (marginal mean = 1,002 ms) than younger (marginal mean = 1,159 ms) adults, and no significant interaction, *F*(1,94) = 3.70, *p* = .058, η_p_^2^ = 0.04 (BF_10_ = 1.07).

#### Priming

**Fig. 7:**
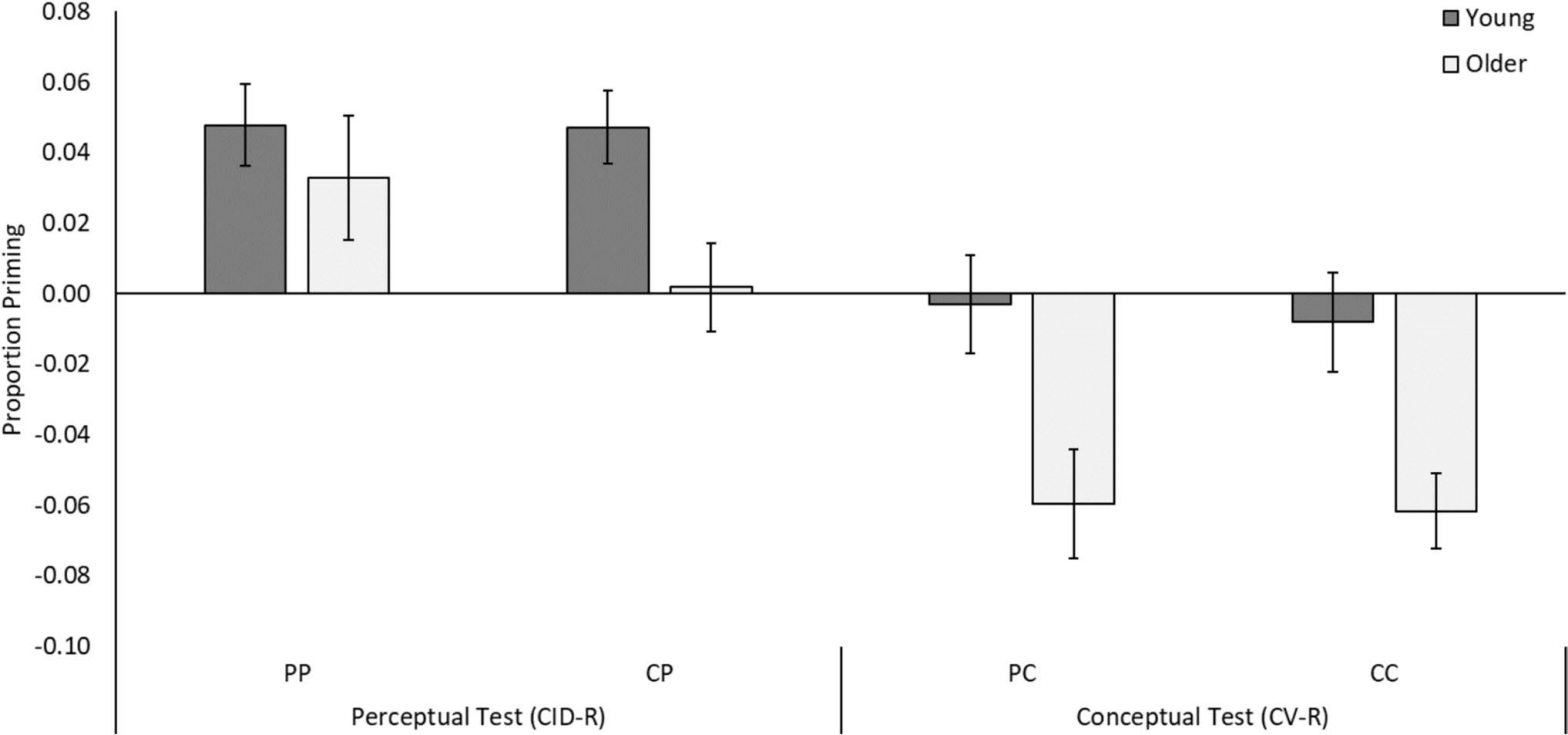
Priming in young and older adults in Experiment 2B (standard error bars)

Trials were screened as described previously, and 524/7680 trials were removed from the CID-R task (young = 203; older = 321), and 319/7680 from the CV-R task (young = 114; older = 205). Priming (Fig. 7, Table 3 for RTs) was once again absent on the CV-R test. A 2 (age) × 2 (encoding type) ANOVA on CID-R priming scores revealed no main effect of encoding type, *F*(1,94) = 2.22, *p* = .140, η_p_^2^ = 0.02 (BF_10_ = 0.43), or age, *F*(1,94) = 3.83, *p* = .053, η_p_^2^ = 0.04 (BF_10_ = 1.18), and no interaction, *F*(1,94) = 2.05, *p* = .156, η_p_^2^ = 0.02 (BF_10_ = 0.52). A-priori planned comparisons of priming in young and older adults revealed a significant difference in the CP condition, *t*(94) = 2.81, *p* = .006, *d* = 0.57, but not the PP condition, *t*(94) = 0.70, *p* = .483, *d* = 0.14 (BF_10_ = 0.27).

#### Recognition

**Fig. 8:**
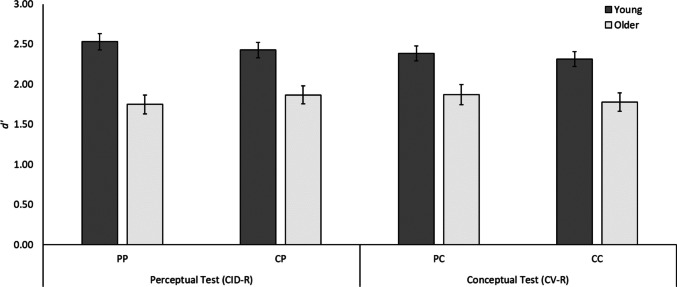
Recognition in young and older adults in Experiment 2B (standard error bars)

On *d’* scores (Fig. 8, Table 4 for a breakdown of responses), there was a main effect of age, *F*(1,94) = 21.61, *p* < .001, η_p_^2^ = 0.19, showing greater recognition in young (marginal mean = 2.42) than older (marginal mean = 1.82) adults, but no main effect of encoding type, *F*(1,94) = 0.76, *p* = .387, η_p_^2^ = 0.01 (BF_10_ = 0.20), or test type,* F*(1,94) = 0.97, *p* = .328, η_p_^2^ = 0.01 (BF_10_ = 0.28), and no interactions: Encoding Type × Age, *F*(1,94) = 1.44, *p* = .234, η_p_^2^ = 0.02 (BF_10_ = 0.32), Test Type × Age, *F*(1,94) = 1.60, *p* = .209, η_p_^2^ = 0.02 (BF_10_ = 0.53), Encoding Type × Test Type, *F*(1,94) = 1.44, *p* = .233, η_p_^2^ = 0.02 (BF_10_ = 0.30), Encoding Type × Test Type × Age, *F*(1,94) = 2.78, *p* = .099, η_p_^2^ = 0.03 (BF_10_ = 0.88).

#### Awareness questionnaire

Eleven young and four older adults were deemed aware in the identification priming task, and eight young and four older adults in the category verification priming task. Given the low levels of awareness, no statistical comparisons were conducted (see Supplementary Materials Table SM2 for means).

### Experiments 2 A and 2B combined analysis

We conducted a combined analysis of priming (CID-R task only) and recognition scores from Experiments 2A and 2B with between-subjects factor Experiment to determine any differences between the data gathered online versus in-person. The 2 (age) × 2 (encoding type) × 2 (experiment) ANOVA on priming revealed a significant main effect of age, *F*(1,188) = 10.29, *p* = .002, η_p_^2^ = 0.05, with greater priming in young (marginal mean = 0.053) than older (marginal mean = 0.015) adults, and the Age × Encoding-Type interaction approached significance, *F*(1,188) = 3.67, *p* = .057, η_p_^2^ = 0.02 (BF_10_ = 1.08). There was no main effect of encoding type, *F*(1,188) = 0.16, *p* = .690, η_p_^2^ < .001 (BF_10_ = 0.12), or experiment, *F*(1,188) = 0.06, *p* = .815, η_p_^2^ < .001 (BF_10_ = 0.16), and no other interactions, Encoding Type × Experiment, *F*(1,188) = 2.57, *p* = .111, η_p_^2^ = .013 [BF_10_ = 0.45]; Age × Experiment, *F*(1,188) = 0.42, *p* = .516, η_p_^2^ = .002 [BF_10_ = 0.25]; Encoding-type × Age × Experiment, *F*(1,188) < 1, *p* = .995, η_p_^2^ < .001 [BF_10_ = 0.21]. Planned comparisons of priming in young and older adults revealed a significant difference in the CP condition, *t*(190) = 3.71, *p* < .001, *d* = 0.15, but not in the PP condition, *t*(190) = 1.60, *p* = .111, *d* = 0.23 (BF_10_ = 0.51).

On recognition (*d′*), there was a main effect of age, *F*(1,188) = 10.79, *p* < .001, η_p_^2^ = .05, with greater recognition in young (marginal mean = 2.16) than older (marginal mean = 1.83) adults, a main effect of encoding type, *F*(1,188) = 26.01, *p* < .001, η_p_^2^ = .12, with greater recognition following conceptual (marginal mean = 2.08) than perceptual (marginal mean = 1.92) encoding, and a main effect of test type, *F*(1,188) = 8.85, *p* = .003, η_p_^2^ = .05, with greater recognition on the CV-R (marginal mean = 2.08) than the CID-R (marginal mean = 1.92) task. There was also a main effect of experiment, *F*(1,188) = 5.44, *p* = .021, η_p_^2^ = .03, showing greater recognition in Experiment 2B (marginal mean = 2.15) than in Experiments 2A (marginal mean = 1.88), and a number of interactions with Experiment, reflecting main effects in Experiments 2A (reported above) that were not present in Experiment 2B, or vice versa: Encoding Type × Experiment, *F*(1,188) = 38.80, *p* < .001, η_p_^2^ = .17 (main effect of encoding type in Experiments 2A only), Test Type x Experiment, *F*(1,188) = 16.24, *p* < .001, η_p_^2^ = .08 (main effect of test type in Experiments 2A only), Age × Experiment, *F*(1,188) = 6.74, *p* = .010, η_p_^2^ = .04 (main effect of age in Experiment 2B only). There was an Encoding-Type × Test-Type interaction, *F*(1,188) = 23.82, *p* < .001, η_p_^2^ = .11, with greater recognition following conceptual (marginal mean = 2.06) than perceptual (marginal mean = 1.64) encoding on the CID-R task, *F*(1,190) = 41.39, *p* < .001, η_p_^2^ = .18, but not the CV-R task *F*(1,190) = 0.71, *p* = .401, η_p_^2^ < .01, but a three-way Encoding-Type × Test-Type × Experiment interaction, *F*(1,188) = 10.42, *p* = .001, η_p_^2^ = .05, revealed that this effect was not present in Experiment 2B. Finally, there was an Encoding-Type × Test-Type × Age interaction, *F*(1,188) = 4.18, *p* = .042, η_p_^2^ = .02, revealing that the effect of greater recognition following conceptual than perceptual encoding on the CID-R task was larger in older adults, *t*(95) = 6.25, *p* < .001, *d* = 0.64, than in young adults, *t*(95) = 2.83, *p* = .006, *d* = 0.29. All other interactions were non-significant, Age × Encoding Type, *F*(1,188) = 3.21, *p* = .075, η_p_^2^ = .02 [BF_10_ = 0.64]; Encoding Type × Age × Experiment, *F*(1,188) = 0.06, *p* = .806, η_p_^2^ < .001 [BF_10_ = 0.24]; Test Type × Age, *F*(1,188) < 1, *p* = .982, η_p_^2^ < .001 [BF_10_ = 0.12]; Test Type × Age × Experiment, *F*(1,188) = 1.78, *p* = .183, η_p_^2^ = .01 [BF_10_ = 0.59]; Encoding Type × Test Type × Age × Experiment, *F*(1,188) = 0.06, *p* = .803, η_p_^2^ < .001 [BF_10_ = 0.15]).

### Discussion

Once again there was no priming on the CV-R task. The main effect of Age on CID-R priming was close to significance, so to clarify we conducted a combined analysis of the priming data from Experiments 2A and 2B, revealing a significant age effect. The Age × Encoding-Type interaction approached significance and Bayes factor analysis provided evidence in favour of an interaction. Priming was greater in young than older adults only following conceptual encoding (i.e., in the CP but not the PP condition). There was no main effect of experiment, indicating compatibility of the priming data collected from the online and in-person experiments.

Priming in the CP condition was greater in Experiment 1 than in Experiments 2A and 2B. This may be because in Experiment 1 participants completed two encoding-test blocks (i.e., a perceptual encoding phase followed by a CID-R test, and a conceptual encoding phase followed by a CID-R test, counterbalanced), whereas in Experiments 2A and 2B participants completed both perceptual and conceptual encoding blocks (counterbalanced) prior to CID-R and CV-R tests. Thus, double the number of items were encoded at the start of the experiment prior to testing in Experiments 2A and 2B (i.e., all 80 items rather than 40 in two encoding-test blocks), so there was a longer study–test delay compared with Experiment 1, and perhaps more interference. This may explain the smaller priming effect in the CP condition in Experiments 2A and 2B compared with Experiment 1, but it is unclear why the same was not observed in the PP condition.

In Experiments 2B recognition was greater in young than older adults and did not vary between the CID-R and CV-R tests or as a function of processing at encoding. The older participants in Experiments 2B were older on average than those in Experiments 2A, which might explain why a significant age effect emerged only in the latter. Given the progressive decline in explicit memory with age (reviewed in Kausler, 1994; Light et al., 2000; Spaan et al., 2003), one would expect the magnitude of the age difference to increase in line with the age of the older participants. The combined analysis of recognition scores from Experiments 2A and 2B revealed main effects of age (young > older), encoding type (conceptual > perceptual), and test type (CV-R > CID-R), but also revealed a main effect of experiment (Experiments 2B > Experiments 2A), in addition to several interaction effects. Caution should therefore be applied in interpreting the combined recognition data, and we return to this in the General Discussion.

## General discussion

Three experiments systematically investigated how processing interacts with age differences in priming. Experiment 1 (online) manipulated perceptual/conceptual encoding, and priming (perceptual identification) was greater in young than older adults when encoding was conceptual. Experiments 2A (online) and 2B (in-person) manipulated perceptual/conceptual encoding prior perceptual (perceptual identification) and conceptual (category verification) priming tests. There was no priming on the category verification task, but perceptual priming was reliably greater in young than older adults following conceptual encoding. In all experiments, there were no age differences in priming when encoding was perceptual.

### Age differences in priming as a function of encoding

As reviewed earlier, prior attempts to understand how normal aging affects implicit memory have produced conflicting results, and this may at least in part be due to differences between studies in cognitive processing requirements. However, the present study is among the first to have directly manipulated processing to examine this. This new evidence suggests that age differences in priming are determined by processing at encoding—apparent following conceptual encoding and absent following perceptual encoding. The underlying mechanisms, however, are yet unknown. In the Introduction we discussed evidence that semantic processing abilities decline with age (see meta-analysis by Hoffman & Morcom, 2018; systematic review by Joyal et al., 2022) and reasoned that reduced priming in older adults for items initially presented in a conceptual encoding task may be explained by inefficient semantic processing of those items, while intact perceptual processing (e.g., Rybash, 1996) in aging explains instances of ‘preserved’ priming for perceptually encoded items (processing deficit hypothesis, Eysenck, 1974). Interestingly, the present age difference in priming following conceptual encoding occurred despite no clear semantic processing deficit in older adults in the encoding phases: In Experiment 1 young adults were more accurate than older adults in both the conceptual and perceptual encoding phases and RTs did not differ between groups. In Experiments 2A older adults were more accurate than young adults in the perceptual encoding block, but there was no age difference in the conceptual encoding block. Both groups made quicker judgements in the conceptual than the perceptual encoding block, but this effect was larger in young adults. In Experiments 2B both groups were less accurate and slower to respond in the perceptual than the conceptual encoding block, and while young adults were generally more accurate than older adults, they were also slower. Thus, another possibility is that older adults were relatively unimpaired in their semantic encoding, but the effects of this processing may have been short-lived compared to young adults, leading to the reduction in priming for conceptual items. An important next step for future studies is to uncover the mechanisms by which conceptual versus perceptual encoding gives rise to age differences in priming.

On a broader level, it will also be important for future studies to more clearly establish the nature of preserved perceptual processing in aging, especially considering the growing evidence that age-related sensory decline is associated with cognitive decline (e.g., Livingston et al., 2020). The present study used visually presented stimuli, and given that visual impairment broadly affects cognitive function (Nagarajan et al., 2022), we specified normal/corrected vision as an eligibility requirement for all participants. Visual impairment may place a greater mental strain on participants by hindering their ability to see and interpret stimuli, affecting encoding. But generally, more work is needed to establish how aging affects different aspects of perceptual cognitive processing. There is a wealth of evidence that performance on perceptual tasks involves posterior cortical areas responsible for processing surface-perceptual features, which are typically spared in neurological aging (e.g., Fleischman et al., 1995; Gabrieli et al., 1994, 1995; Keane et al., 1991; Squire, 1994). Nevertheless, while basic perceptual abilities like detection and discrimination show little decline, there is some evidence for a decline in more complex perceptual processes like temporal integration (Roberts & Allen, 2016).

### Perceptual versus conceptual priming

While priming was robust on the continuous identification (CID, perceptual) task, it was largely absent on the category verification (CV, conceptual) task. These tests were selected based on their established processing requirements, and because it was possible to match them closely on all characteristics except processing. For example, both involved a speeded task with an RT measure, and within-trial events and durations were consistent. However, there are a range of other perceptual and conceptual priming tasks, which vary greatly in their characteristics (see Blaxton, 1989; Tulving & Schacter, 1990). Perceptual tasks such as perceptual identification, word-stem completion (WSC), and word-fragment completion (WFC) evoke perceptual processing of items. The CID task, and other such speeded perceptual measures, are generally considered to be among the most clean and sensitive. The goal—to identify items as quickly as possible—is clear and rigid, and does not encourage participants to adopt different strategies. Response variability is therefore typically lower in comparison to other perceptual tasks like WSC, in which the goal—to complete stems with the first word that comes to mind—is less rigid and participants have more time. This issue was discussed in detail by Buchner and Wippich (2000), who demonstrated greater statistical reliability of a perceptual identification task compared to WSC, and greater sensitivity to age differences. A number of studies have reported reliable reductions with age on perceptual priming tasks (e.g., Abbenhuis et al., 1990; Chiarello & Hoyer, 1988; Keane et al., 2004; Russo & Parkin, 1993; Small et al., 1995; Soldan et al., 2009; Ward et al., 2013b, 2015, 2020; Wiggs & Martin, 1994), but there are also instances of age equivalent priming (e.g., Henson et al., 2016; Jelicic et al., 1996; Light et al., 1992; Light & Singh, 1987; Mitchell & Bruss, 2003; Park & Shaw, 1992; Spaan & Raaijmarkers, 2010; Sullivan et al., 1995; Wiggs et al., 2006). It must be cautioned that null age differences, especially on tasks with low reliability, may reflect a failure to statistically detect small but genuine differences, especially if sample sizes were insufficient—a problem in several studies that pre-date the move towards sample size calculations and pre-registration. All adequately powered studies using the CID task with conceptual encoding have uncovered a significant age-related reduction in priming (reviewed in Ward & Shanks, 2018; note that there are instances of age-invariant CID priming in amnesia, e.g., Conroy et al., 2005).

In contrast, conceptual priming tasks draw upon semantic processing. Most involve presenting cues that are conceptually related to studied items. For example, in a category exemplar generation (CEG) task participants are presented with categories (e.g., animals, vehicles), some relating to previously studied items, and asked to generate examples of items. Priming occurs if more previously studied than new items are produced. There are examples of reduced priming with age on these types of accuracy-based conceptual tasks (e.g., Jelicic et al., 1996; Maki et al., 1999; Stuart et al., 2006), but other studies have reported no age difference (e.g., Isingrini et al., 1995; Light et al., 2000; Mitchell & Bruss, 2003). All except Mitchell and Bruss (2003) involved conceptual encoding (pleasantness ratings), with items presented for 2–5 s, and sample sizes were similar (20–24 participants per group), so the reasons behind the discrepancies are unclear. Generally, the CEG task does not appear to be a particularly robust or sensitivity measure of conceptual priming, and there is also evidence that it may be susceptible to explicit contamination (Light & Albertson, 1989; Maki & Knopman, 1996). Like CID, the CV task captures RT-based priming for previously studied items, but the decision—judging whether an object is an instance of a particular category—involves conceptual processing (Light et al., 2000). Its apparent inability to detect priming in the present experiments, however, raises concerns over its reliability. Given robust recognition, the lack of priming cannot be explained by insufficient encoding; it must be due to the CV task itself. In the paragraphs that follow we review studies that have used this task to examine conceptual priming in aging to pinpoint a suitable procedure for its use in future studies.

In Ward (2023), young and older participants studied words in perceptual and conceptual encoding blocks prior to a CV task. On each CV trial a word (studied/new) was presented in centre screen and a category name simultaneously above, and participants made a yes/no key-press response (RT captured) to indicate whether the word matched the category. There was no priming in older adults (.001 following perceptual encoding; <.001 following conceptual encoding), but priming was significantly above zero in young adults following perceptual encoding (.042; .021 following conceptual encoding). In Light et al. (2000), participants studied words under full attention (FA) and divided attention (DA) prior to a CV task (Experiments 2 and 3). Words appeared above category names on the screen, simultaneously in Experiment 2, but in Experiment 3 the category name was presented for 1 s before the target word. In their Experiment 2, priming was below zero in older adults (−.084 and −.095 in FA and DA, respectively), and significantly above zero in young adults (.066 in both FA and DA conditions). However, in their Experiment 3 priming occurred in both age groups (young: FA: .072, DA: .061; older: FA: .055, DA: .028). One may take from this that presenting category names prior to targets increases priming, but it should be noted that in Light et al.’s Experiment 2 there was one encoding phase and one CV test, separated by a 7-min filler task, while in their Experiment 3 there were two study–test blocks and no filler task. It is impossible to know which of these differences, or combination, gave rise to the different experimental outcomes, contributing to an age difference in priming in Experiment 2, but no age difference in Experiment 3. Gabrieli et al. (1999) also used a CV task (Experiment 3), but their study compared individuals with Alzheimer’s disease (AD) with healthy controls. Participants made category verifications at both encoding and test, and the procedure once again differed: Categories were presented for 4 s prior to target words, and participants responded verbally. Priming was above zero in healthy older adults (59 ms) and AD patients (76 ms), and not significantly different between groups. A similar procedure was used by Vaidya et al. (1997), involving only young adults. In their study, priming was above zero in shallow (.031) and deep (.042) processing conditions (Experiment 5), and auditory (.059) and visual (.067) conditions (Experiment 6).

Thus, minor procedural differences make a big difference to the measurement of priming on the CV task. It is difficult to pinpoint an ‘ideal’ CV-task procedure, and it should be noted that the present study is the first to use object stimuli, but presenting categories prior to targets appears to be beneficial, especially for older adults. Presenting categories and targets simultaneously may produce longer RTs and/or greater variability because there is more to process at once, which could wash out priming. Simultaneous presentation explains the lack of priming in studies that have used this approach, and may have contributed to the negative priming scores observed in older adults in Light et al.’s Experiment 2 (−.084 and −.095 in FA and DA conditions, respectively), and the present Experiment 2B (PC= −.060; CC= −.062). Light et al. (2000) pointed out that presenting categories prior to targets ensures that categories are processed first, and eliminates processing time for categories from decision latencies. Indeed, there are no cases of negative priming under these such sequential presentation conditions. There are other factors that may have also contributed to negative priming, for instance, it could indicate interference between judgements at encoding and test. If we consider an example where the item ‘camera’ is presented at encoding and participants correctly judge as manufactured. If at test ‘camera’ is re-presented but participants are asked to decide if it is a ‘type of food’, then assuming that ‘camera’ is still linked to concept ‘manufactured’, there may be a clash with the concept ‘food’, slowing down the response. In other words, competing categories are being activated for the same item at encoding and test (e.g., Mayr & Buchner, 2007). Given all of this, on a broader level, one could consider that conceptual judgements may be less compatible with a speeded measure; that is, attempting to make fast category decisions may be more challenging than identifying items as quickly as possible. Simply presenting a previously studied item at test may not automatically evoke its category affiliation, this appears to be slower and require more effort.

Clearly, more work is needed to disambiguate the effects of aging on conceptual priming, with efforts concentrated on identifying suitable tasks. To utilise the CV task for this purpose, future studies need to establish the optimal procedure by formally comparing simultaneous versus sequential presentation of categories and targets, and different types of stimuli. Beyond this, the matter of reliability and sensitivity of conceptual priming tasks is an important issue that calls for extensive systematic review. More efforts have been concentrated on perceptual measures in this regard, and perceptual identification is generally considered a robust task that is sensitive to age differences (see Buchner & Wippich, 2000; Ward et al., 2013a, b). However, the field calls for a review of all studies, segregating based on processing requirements, encoding, type of stimuli, and more.

### Effects on recognition

The age and processing effects on priming in Experiment 1 were mirrored in the recognition data—recognition was significantly greater in young than older adults only following conceptual encoding. However, the recognition outcomes in Experiments 2A and 2B were mixed—there was a reliable age difference in Experiments 2B but not Experiments 2A, which may reflect the greater average age of the older participants in Experiments 2B (76.29 years compared with 69.19 years in Experiments 2A). In Experiments 2A, like in Experiment 1, recognition was greater following conceptual than perceptual encoding on the CID-R test, but there was no effect of encoding on recognition in Experiments 2B. To clarify this, a combined analysis of recognition in Experiments 2A and 2B was conducted, revealing lower recognition in older than younger adults, in the perceptual than the conceptual encoding condition, and in the CID-R than the CV-R test. The effects of age and encoding are as would be expected, but the difference in recognition between the CID-R and CV-R tests suggests that the type of priming task may have somehow influenced recognition judgements, because the recognition task was identical in both cases. Caution should be applied in the interpretation of effects from this combined analysis because recognition varied as a function of factor Experiment, and there were several higher-order interactions with Experiment, but nevertheless it will be important for future studies to examine the possibility of such ‘implicit contamination’.

The concurrent method of assessing priming and recognition is beneficial as it allows the two to be captured within a few hundred milliseconds of one another for every test item, making them ideally suited for comparison. There is a wealth of evidence that the identification task in the CID-R procedure is not susceptible to explicit contamination (e.g., Brown et al., 1991, 1996; Ward et al., 2013b), possibly because identification is accomplished too quickly for the engagement of effortful memory strategies (MacLeod, 2008). Brown et al. (1996) and Ward et al. (2013b) found no difference in priming when identification and recognition were measured concurrently trial-by-trial compared with separate experimental phases, and there were no differences in priming between participants who were aware versus unaware of the fact that items from the encoding phase were re-presented. In the present experiments, we found very low levels of awareness. The highest levels were in Experiment 2A where 19/48 young adults were aware of the purpose of the identification task. However, this did not affect priming or interact with processing. There is some evidence that explicit strategies can affect conceptual priming in older adults (e.g., Mitchell & Perlmutter, 1986), but this is unlikely to have affected the present study given such low levels of awareness. However, the broader question of whether conceptual priming tasks are generally susceptible to explicit contamination has not been fully addressed. As noted above, several studies have directly manipulated test awareness to examine the effect on perceptual priming, but similar studies are needed to uncover the effect on conceptual priming.

### Theoretical implications

The present findings have implications for theories of cognitive aging and the organisation of memory. While it was once widely believed that implicit memory is preserved in normal aging despite a reduction in explicit memory (reviewed in Fleischman, 2007; Mitchell & Bruss, 2003), this has been fundamentally challenged in recent years. There is growing evidence that implicit memory is not completely spared with age (reviewed in Ward & Shanks, 2018), and the present findings suggest that age differences in priming are real but vary depending on the manner of processing. Further, preserved priming coupled with reduced explicit memory in older compared with younger adults has often been cited as evidence for functionally distinct memory systems (e.g., Gabrieli, 1998, 1999; Mitchell, 1989; Mitchell et al., 1990; Schacter, 1987; Schacter & Tulving, 1994; Squire, 1994, 2004, 2009; Tulving & Schacter, 1990), but the growing evidence that priming is not immune to age-related decline is more consistent with the view that explicit and implicit memory are driven by a single underlying system (e.g., Berry et al., 2006, 2008a, b, 2012; Buchner & Wippich, 2000; Nosofsky et al., 2012).

### Online versus in-person studies

Another goal of the present study was to provide the first direct comparison of online versus laboratory experiments on priming in aging. As noted earlier, online experimental research is rapidly gaining popularity and provides an excellent way of accessing large and diverse samples. We used online platform Gorilla in Experiment 1 and 2A, taking precautions to maximize the quality of the data and standardize the setting. Experiment 1 (online) corroborated the findings of Ward (2024), which was conducted in person. Experiment 2B (in person) was a direct replication of Experiment 2A (online), and a combined analysis of priming revealed no effect of Experiment, suggesting compatibility of data collected online and in the laboratory. However, recognition differed as a function of the mode of testing, with greater performance in the laboratory (Experiment 2B) than online (Experiment 2A), perhaps reflecting greater motivation or effort when participants are tested in person. It could be that small differences in motivation or effort, which, if present, do not affect priming, influence more demanding tasks like recognition. Greene and Naveh-Benjamin (2022) also suggested that online studies may include more ‘low-effort’ participants, and that exclusion rates are typically greater in online studies, reflecting a higher proportion of participants failing to meet specific performance thresholds. Indeed, exclusion rates were higher in the present Experiment 1 and 2A (online) compared with Experiment 2B (in person). In these experiments excluded participants were replaced, but potential differences between online and in-person samples is an important general consideration for online experimental research.

## Conclusion

Three experiments uncovered an age difference in perceptual identification priming favouring young adults when encoding was conceptual, but no age difference following perceptual encoding. This suggests that implicit memory may not be wholly preserved with age, but age differences emerge as a function of the way stimuli are processed during encoding. The study adds significantly to the growing body of evidence of the feasibility of online experimental research in the field of cognitive aging.

## Supplementary Information

Below is the link to the electronic supplementary material.

Supplementary file1 (DOCX 26 KB)

## Data Availability

The de-identified raw data and analysis files are available online (https://osf.io/t7b2y/). Materials (object stimuli) are freely available at the Bank of Standardized Stimuli (Brodeur et al., 2014).

## Footnotes

1 In-person experiments involving healthy older participants usually include screening for cognitive impairment. The Mini Mental State Exam (MMSE; Folstein et al., 1975) is a popular tool; however, this is not suitable for self-administration through online testing. Participants in Experiment 1 completed the Early Dementia Questionnaire (EDQ; Arabi et al., 2013) electronically. Previously used in Ward (2023), this was deemed suitable for self-administration. The EDQ consists of 20 statements that participants respond to on a Likert scale, taking into account the previous 2 years (e.g., ‘require checklist as memory support’: 0 = *never*, 1 = *seldom*, 2 = *sometimes*, 3 = *always*). The numbers 0–3 are summed to give an overall score (0–60), and a score ≥8 indicates possible impairment. We intended to only include participants scoring <8 in the final sample, but 22 scored below this threshold and were not cognitively impaired, suggesting that at least some participants failed to understand the EDQ items. All participants confirmed in their correspondence with the researcher that they had no concerns with their cognitive function and no diagnosis of cognitive impairment or dementia. This was also confirmed during consent, where participants ticked boxes to indicate that they met the eligibility criteria (no cognitive impairment/dementia was explicitly stated). Further, there were no outliers in the data to suggest abnormal memory function. Given this, the decision was made not to exclude participants in Experiment 1 based on their EDQ score. No EDQ was administered in Experiment 2A, but additional screening questions were added, and the MMSE was used in Experiment 2B (in-person).

2 Accuracy in the conceptual encoding condition in all experiments was based on the correct answer (i.e., natural/manufactured item); however, accuracy in the perceptual encoding condition in Experiments 1 and 2A was based on the model response, in view that the tilted/upright judgements may be somewhat subjective. Although accuracy was defined differently in the two conditions, this is unlikely to have influenced the encoding phase results, particularly because the modal response of participants in the conceptual condition also matched the correct answer. In Experiment 2B the perceptual encoding-phase judgement was changed to angular/rounded, and the correct answer was determined by classifications made by a separate sample of volunteers (see Method of Experiment 2B).

3 Prolific is an online recruitment service (https://www.prolific.co/). It has been shown to produce high-quality data, superior to many other platforms (e.g., Peer et al., 2017).

4 The preregistered encoding-phase accuracy threshold was relaxed from 70% to 60% across blocks in Experiment 2A. This was because accuracy was lower than anticipated based on Experiment 1 (Experiment 1 final sample range = 71–95% correct, Experiment 2A final sample range = 60–95% correct), and a high proportion of participants in Experiment 2A failed to meet the 70% threshold despite good performance in the rest of the experiment, exceeding all other performance thresholds. For Experiment 2B, we preregistered an encoding-phase accuracy threshold of 60%, but this was also slightly relaxed (final sample range = 56–94%), as, again, some participants scored below this threshold despite exceeding all other performance thresholds. Careful consideration was given to these decisions, and we reasoned that although the encoding-phase judgements were not complex, encouraging participants to respond as quickly as possible may have contributed to a high volume of erroneous key presses. Indeed, in Experiment 2B (in person), several participants reported pressing the wrong key despite knowing the correct answer, and this may have also occurred in Experiment 2A (online so no informal participant feedback). All other preregistered performance thresholds were adhered to, replacing participants where these were not met.
