# Supplementary material for: Conceptual but not perceptual encoding leads to age differences in priming
Source: Mem Cognit. 2026 Apr 1;54(6):2341–64. doi: 10.3758/s13421-026-01873-7 (PMC13461660; doi:10.3758/s13421-026-01873-7)
Supplement: Supplementary file 1 [file 13421_2026_1873_MOESM1_ESM.docx]

**Supplementary Materials**

**Analysis of recognition response bias**

Response bias was calculated in each Experiment as C = -0.5*(zHits-zFA), where scores of C>0 indicate a conservative bias, a tendency to respond ‘no’ (new), and scores of C<0 indicate a liberal bias, a tendency to respond ‘yes’ (old). See Supplementary Materials Table SM3 for scores.

A 2(Age) x 2(Encoding-type) ANOVA on C scores in Experiment 1 revealed a main effect of Age, *F*(1,68)=6.27, *p*=.015, η_p_^2^=.08, but no main effect of Encoding-type, *F*(1,68)=0.40, *p*=.528, η_p_^2^=.01, and no Age x Encoding-type interaction, *F*(1,68)=0.81, *p*=.371, η_p_^2^=.01. Older adults (marginal mean=-0.82) were more liberal than young adults (marginal mean=-0.27). A 2(Age) x 2(Encoding-type) x 2(Test-type) ANOVA in Experiment 2A revealed a main effect of Age, *F*(1,94)=11.48, *p*=.001, η_p_^2^=.11, with a tendency towards liberal responding in young adults (marginal mean=-0.17), but a tendency towards conservative responding in older adults (marginal mean=0.16). There was also a main effect of Encoding-type, *F*(1,94)=55.18, *p*<.001, η_p_^2^=.37, with more liberal responding in the conceptual condition (marginal mean=-0.10) and more conservative responding in the perceptual condition (marginal mean=0.09), and a main effect of Test-type, *F*(1,94)=8.96, *p*=.004, η_p_^2^=.09, with more liberal responding on the CID-R task (marginal mean=-0.11) and more conservative responding on the CV-R task (marginal mean=0.10). However, there was an Age x Test-type interaction, *F*(1,94)=7.75, *p*=.006, η_p_^2^=.08, indicating more liberal responding in older than young adults on the CID-R task (*t*(94)=4.85, *p*<.001, *d*=0.99 and *t*(94)=4.41, *p*<.001, *d*=0.91, in the PP and CP conditions, respectively), but no age difference on the CV-R task (*t*(94)=1.07, *p*=.288, *d*=0.22 and *t*(94)=0.99, *p*=.323, *d*=0.20, in the PC and CC conditions, respectively). There was also an Encoding-type x Test-type interaction, *F*(1,94)=31.26, *p*<.001, η_p_^2^=.25, indicating and more liberal responding in the CP than the PP condition (*t*(47)=5.25, *p*<.001, *d*=0.76 and *t*(47)=8.35, *p*<.001, *d*=1.21, for young and older adults, respectively), but no difference between the PC and CC conditions (*t*(47)=1.40, *p*=.168, *d*=0.20 and *t*(47)=2.03, *p*=.048, *d*=0.29, for young and older adults, respectively [Bonferroni corrected alpha value of .013]). There was no Age x Encoding-type interaction, *F*(1,94)=1.78, *p*=.185, η_p_^2^=.02, or Age x Encoding-type x Test-type interaction, *F*(1,94)=1.57, *p*=.213, η_p_^2^=.02.

Despite these differences in Experiment 2A, participants in Experiment 2B were generally neutral to conservative in their responding, and the ANOVA revealed no significant main effects or interactions: Age, *F*(1,94)=3.39, *p*=.068, η_p_^2^=.04, Encoding-type, *F*(1,94)=0.77, *p*=.384, η_p_^2^=.01, Test-type, *F*(1,94)=0.29, *p*=.590, η_p_^2^<.01, Age x Encoding-type, *F*(1,94)=1.51, *p*=.223, η_p_^2^=.02, Age x Test-type, *F*(1,94)=1.17, *p*=.282, η_p_^2^=.01, Encoding-type x Test-type, *F*(1,94)=1.52, *p*=.219, η_p_^2^=.02, Age x Encoding-type x Test-type, *F*(1,94)=2.75, *p*=.101, η_p_^2^=.03.

**Table SM1**

*Number and organisation of items between experimental phases*

| Experiment 1 | Block 1 | Encoding (perceptual) | 40 |
| --- | --- | --- | --- |
|  |  | Test (CID-R) | 80 (40 old [perceptual], 40 new) |
|  | Block 2 | Encoding (conceptual) | 40 |
|  |  | Test (CID-R) | 80 (40 old [conceptual], 40 new) |
| Experiments 2A / 2B | Encoding | Block 1 (perceptual) | 40 |
|  |  | Block 2 (conceptual) | 40 |
|  | Test | Block 1 (CID-R) | 80 (40 old [20 perceptual, 20 conceptual], 40 new) |
|  |  | Block 2 (CV-R) | 80 (40 old [20 perceptual, 20 conceptual], 40 new) |

*Note.* In all experiments, the order of the blocks was counterbalanced. In this depiction, the perceptual conditions are presented first, but half of the participants began with the conceptual condition.

**Table SM2**

*Proportion priming in aware versus unaware participants in Experiments 1, 2A and 2B*

|  | Aware | Unaware |
| --- | --- | --- |
|  | *M* (*SD*) | *M* (*SD*) |
| Experiment 1 |  |  |
| Perceptual block |  |  |
| Young | -0.06 (0.05) | -0.05 (0.09) |
| Older | -0.08 (0.09) | -0.05 (0.07) |
| Conceptual block |  |  |
| Young | 0.24 (0.05) | 0.21 (0.09) |
| Older | 0.14 (0.05) | 0.10 (0.06) |
| Experiment 2A  Perceptual priming – CID-R test |  |  |
| CP Young | 0.03 (0.10) | -0.09 (0.11) |
| PP Young | 0.03 (0.08) | -0.05 (0.08) |
| CP Older | -0.03 (0.12) | 0.02 (0.12) |
| PP Older | -0.03 (0.08) | 0.02 (0.10) |
| Conceptual priming – CV-R test |  |  |
| CC Young | -0.06 (0.14) | 0.01 (0.17) |
| PC Young | -0.03 (0.08) | -0.01 (0.18) |
| CC Older | -0.01 (0.15) | -0.01 (0.07) |
| PC Older | -0.01 (0.09) | -0.01 (0.08) |
| Experiment 2B  Perceptual priming – CID-R test |  |  |
| CP Young | 0.04 (0.10) | 0.05 (0.06) |
| PP Young | 0.04 (0.08) | 0.05 (0.08) |
| CP Older | -0.02 (0.15) | 0.00 (0.08) |
| PP Older | 0.05 (0.11) | 0.03 (0.12) |
| Conceptual priming – CV-R test |  |  |
| CC Young | -0.03 (0.10) | -0.00 (0.10) |
| PC Young | 0.01 (0.08) | -0.01 (0.10) |
| CC Older | 0.01 (0.06) | -0.07 (0.07) |
| PC Older | -0.02 (0.05) | -0.06 (0.11) |

*Note.* CC = conceptual encoding—conceptual test; PC = perceptual encoding—conceptual test; CP = conceptual encoding—perceptual test; PP = perceptual encoding—perceptual test.

**Table SM3**

*Recognition task response bias (C) in Experiments 1, 2A and 2B*

|  | Young | Older |
| --- | --- | --- |
|  | *M* (*SD*) | *M* (*SD*) |
| Experiment 1 |  |  |
| Perceptual encoding | -0.29 (0.80) | -0.75 (1.21) |
| Conceptual encoding | -0.26 (0.82) | -0.89 (1.08) |
| Experiment 2A |  |  |
| PP | -0.25 (0.69) | 0.32 (0.44) |
| CP | -0.48 (0.60) | -0.02 (0.40) |
| PC | 0.06 (0.79) | 0.21 (0.51) |
| CC | 0.00 (0.73) | 0.12 (0.50) |
| Experiment 2B |  |  |
| PP | -0.03 (0.45) | 0.24 (0.56) |
| CP | 0.02 (0.40) | 0.18 (0.57) |
| PC | 0.00 (0.39) | 0.10 (0.65) |
| CC | 0.04 (0.41) | 0.15 (0.61) |

*Note:* PP: perceptual encoding—perceptual test; CP: conceptual encoding—perceptual test; PC: perceptual encoding—conceptual test; CC: conceptual encoding—conceptual test.
